# Stereoselective mechanochemical synthesis of thiomalonate Michael adducts via iminium catalysis by chiral primary amines

**DOI:** 10.3762/bjoc.20.198

**Published:** 2024-09-12

**Authors:** Michał Błauciak, Dominika Andrzejczyk, Błażej Dziuk, Rafał Kowalczyk

**Affiliations:** 1 Faculty of Bioorganic Chemistry, Wrocław University of Science and Technology, wyb. Wyspiańskiego 27, 50-370 Wrocław, Poland; 2 Current company: PCC EXOL, Poland; 3 Institute of Advanced Materials, Wrocław University of Science and Technology, wyb. Wyspiańskiego 27, 50-370 Wrocław, Poland

**Keywords:** asymmetric catalysis, iminium catalysis, mechanochemistry, organocatalysis, thioesters

## Abstract

The study presents a novel approach utilizing iminium salt activation and mild enolization of thioesters, offering an efficient and rapid synthesis of Michael adducts with promising stereoselectivity and marking a significant advancement in mechanocatalysis. The stereoselective addition of bisthiomalonates **1**–**4** to cyclic enones and 4-chlorobenzylideneacetone proceeds stereoselectively under iminium activation conditions secured by chiral primary amines, in contrast to oxo-esters as observed in dibenzyl malonate addition. Mild enolization of thioesters allows for the generation of Michael adducts with good yields and stereoselectivities. Reactions in a ball mill afford product formation with similar efficacy to solution-phase reactions but with slightly reduced enantioselectivity, yet they yield products in just one hour compared to 24 or even 168 hours in solution-based reactions. It is noteworthy that this represents one of the early reports on the application of iminium catalysis using first-generation chiral amines under mechanochemical conditions, along with the utilization of easily enolizable thioesters as nucleophiles in this transformation.

## Introduction

Mechanochemistry, particularly solventless processes under ball milling conditions, offers the opportunity to devise unconventional reaction pathways [1–5]. The transient impact of kinetic energy, channeled into the reaction, facilitates overcoming the constraints inherent in equilibrium models. Thus, employing limited substrates could potentially yield diverse products through a simple alteration of conditions, compared to solvent-based methods [6–8]. Furthermore, the integration of mechanochemistry and organocatalysis leads to the development of more sustainable transformations, characterized by reduced reaction times, decreased catalyst loadings, and significantly diminished solvent usage and waste production [9–11].

The pioneering studies by Bolm [12–13], and Juaristi et al. [14]. have significantly advanced chiral secondary amine-catalyzed stereoselective reactions under ball milling conditions, representing a widely explored activation mode in mechanochemical-mediated transformations. However, reports on chiral primary iminium [15–16] or iminium-ion catalysis [17] under ball-mill conditions are scarce, in contrast to the abundance of transformations catalyzed by such covalent catalysis.

Among the numerous organocatalytic reactions facilitated by primary amine-based iminium ions, Michael-type additions deserve special attention. The controlled formation of C–C and C–X bonds in a stereoselective fashion has found extensive application in asymmetric synthesis. Notably, the addition of malonates has attracted significant interest, albeit primarily limited to methyl or ethyl diesters [18–20]. The combination of iminium catalysis with hydrogen bonding units has been essential for achieving high reactivity and enantioselectivities [21–22]. Additionally, the reactivity of the nucleophilic addition is influenced by substitutions near the electron-poor double bond. This approach requires 30 mol % of catalyst and a reaction time of two days under a pressure of 0.8 GPa [23].

Less reactive benzyl malonates, which allow for the cleavage of a free carboxylic group without the need for harsh base- or acid-mediated conditions [24], undergo additions catalyzed by primary amines [19]. However, these transformations are hampered by extended reaction times, sometimes up to 168 hours. An intriguing example involves the use of a bifunctional primary amine-sulfonamide catalyst, which activates benzylideneacetone towards dibenzyl malonate, with the presence of water accelerating the reaction [25].

An alternative approach, where enhancing the reactivity of a relatively inert acceptor does not necessarily lead to increased reaction rates, involves the use of more reactive nucleophiles. In this context, varying the stabilization energy of carboxylic acid derivatives by switching from oxoesters to thioesters is the significant acceleration of the reaction progress [26–27]. The crucial enhancement of anion stabilization by deprotonation of the methylene group in bisthiomalonates was hypothesized to surpass that of analogous dibenzyl malonate [28–29].

**Scheme 1:**
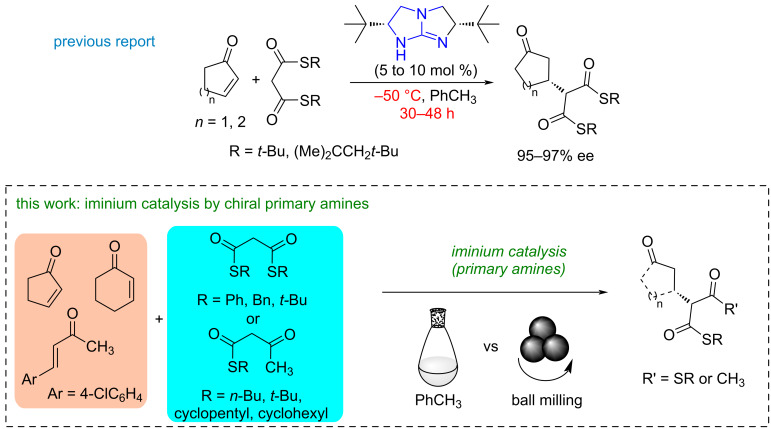
Two examples of base-catalyzed addition of thiomalonates to enones and the scope of the work.

Surprisingly, the employment of NaH, NaSEt, or *t*-BuOLi as relatively strong bases proved ineffective in facilitating the Michael addition of bisthiomalonates to conjugated ketones, whereas DABCO enabled the formation of desired products under mild conditions [30]. However, reports of highly stereoselective protocols utilizing hydrogen bonding catalysis have mainly focused on nitroalkenes or guanidine-mediated addition of bisthiomalonates to maleimides, cyclic enones, and acyclic 1,4-dicarbonylbutenes, albeit at low temperatures and with relatively long reaction times, further limited to a narrow scope of thioesters (Scheme 1) [31].

Recently, we described a cinchona-squaramide-based catalytic system for the highly stereoselective addition of various bisthiomalonates to chalcones and dienones [29]. While recognizing the potential for greater selectivity enhancement and time savings with ball milling, hydrogen-bonding catalysis was effective only with aromatic enones, yielding low conversions and stereoselectivities when applied to benzylidene acetones or cyclic enones (vide infra).

In our latest research in establishing a mild organocatalytic protocol for incorporating benzyl malonates and bisthiomalonates into cyclic enones and benzylidene acetones, we have developed a novel approach based on iminium catalysis employing chiral primary amines under ball milling conditions. Ball milling induces localized and transient temperature and pressure increases which could influence changes in the free activation volume [32–34], favoring Michael additions [35]. Furthermore, condensation reactions, including the formation of transient C=N bonds, are significantly promoted under ball milling conditions [12–14]. It is noteworthy that the application of primary amines for activating cyclic ketones under mechanochemical conditions remains largely unexplored [36]. We aim to fill this research gap by introducing an effective methodology for generating enantiomerically enriched products, thereby expanding the scope of applications and contributing to a deeper understanding of stereoselective covalent catalysis, particularly in the field of mechanochemistry.

## Results and Discussion

**Scheme 2:**
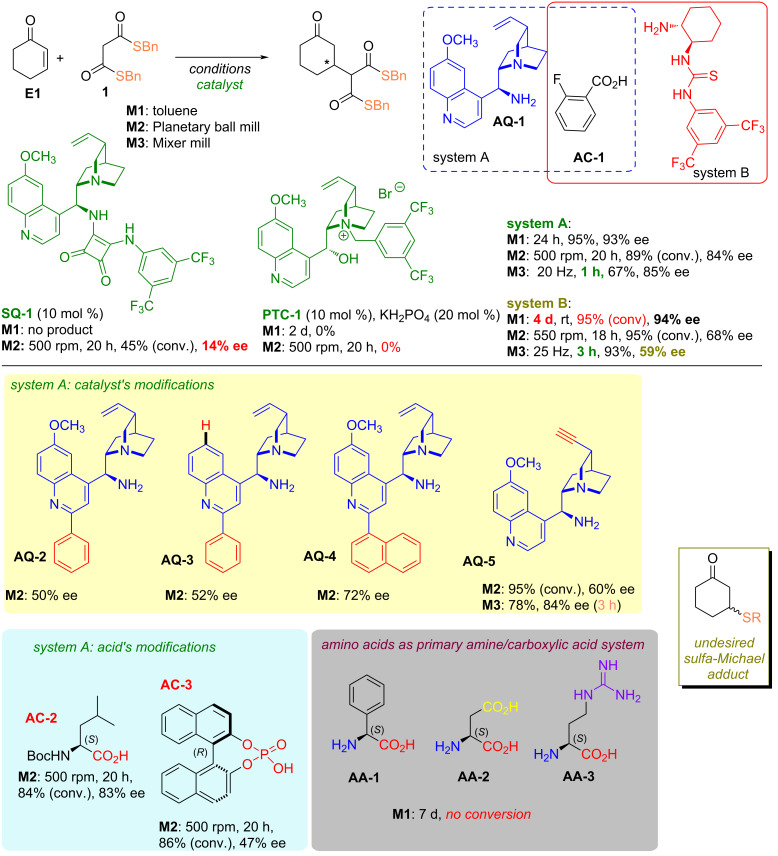
Tested reactions of cyclohexanone with dibenzyl thiomalonate **1**.

Based on our previous experiences with low-reactive Michael acceptors [29], initial attempts were carried out using bifunctional squaramides, derivatives of cinchona. However, up to 45% yield of the desired product was obtained with poor enantioselectivity (14%, Scheme 2). Considering the stability of thioester groups in aqueous environments, we also decided to apply conditions conducive to hydrophobic amplification [37], which unfortunately also did not yield the intended effect. Another approach is to generate and stabilize the active nucleophile form as an anion thus increasing its reactivity by chiral quaternary ammonium salt in the asymmetric variant of the phase-transfer catalysis. Nevertheless, no product was formed despite several conditions being examined. Finally, an attempt to activate the electrophile was performed. We chose iminium salt catalysis particularly employing chiral primary amines for this purpose to enable activation of the enone system. Such a method lowers the energy of the LUMO orbital and subsequently reduces the energy barrier between the nucleophile's HOMO orbital resultingly, expedites the entire transformation [38]. Moreover, the presence of the stereogenic center in proximity to the reactive moiety of the covalent substrate adduct to the catalyst contributes to the efficient transfer of chirality to the resulting product, even in the distant position of the 1,6-conjugated system [39]. For these reasons, iminium catalysis using primary amines appears to be the ideal tool in addition to reactions to cyclic enones and benzylidene acetones. However, our concern was whether the thioester group, whose activity towards nitrogen nucleophiles significantly exceeds that of analogous oxo-esters [26–27,40], would remain unaffected in the presence of a nucleophilic catalyst. Test reactions between cyclohexenone and thiomalonate **1** conducted in toluene at room temperature indicated the formation of the product with high conversions and efficiencies (Scheme 2).

Application of *epi*-aminoquinine (**AQ-1**) in combination with 2-fluorobenzoic acid (system A) led to the product with 93% ee after 24 h, while the bifunctional primary amine-thiourea catalysts (system B) required 4 days to provide an adduct with similar enantioselectivity. Prolonged reaction time is in general the innate nature of organocatalytic reactions employing iminium activation approaches. With the aim to decrease the time along with the generation of a product with an acceptable yield, we decided to perform the reaction under ball milling conditions reasoning that choice by limiting the solvent, increasing the concentration of both reagents and catalyst accompanying with the efficient mixing supported moreover by the local pressure increase. Albeit the transformation performed in the planetary ball mill required 20 h to proceed, the same reaction in the mixer mill offering the greater energy impact by the rise of mixing frequency led to the product after 1 hour with an acceptable yield of isolated product. However, the decrease in the enantioselectivity was noted in comparison to the reaction under standard conditions in toluene (93% vs 85% in ball mill). Surprisingly, the essential loss of enantioselectivity was noted when system B was applied in analogous transformation.

Although an impressive boost of the reaction rate in the ball mill was observed (3 h vs 4 days), the stereochemical outcome suffered giving the desired adduct with 59% ee instead of 94% ee in solution. Owing to the introduction of an additional functional group in the form of a thiourea moiety does not entail a significant enhancement of catalytic system efficiency and results from the dramatic decrease in enantioselectivity observed in mill reactions. Additionally, the catalyst’s synthesis requires an extra synthetic step. We decided to conduct further investigations utilizing system A or its modifications.

First, changes in the *epi*-amino alkaloid core of the two-component catalytic system were investigated. Introduction of the 2’-substitution to the quinine core as in **AQ-2** and **AQ-4** (Scheme 2) resulted in the decrease of chirality transfer providing the product with 50% and 72% ee, respectively. A similar trend was observed when **AQ-3** was applied. Besides, the modifications of the quinuclidine ring through replacing the vinyl substituent in the parent quinine core by a triple bond (**AQ-5**) led to no substantial improvement in the mixer mill, but the decrease of enantioselectivity in the reaction performed in the planetary mill. Further variations of system A concerned the acid’s role. However, no impact of the acid was noted while using the amino acid derivative **AC-2**, but the dramatic loss of selectivity was observed in the case of chiral phosphoric acid’s application **AC-3**. Thus, it is rather unlikely the iminium-ion catalysis to operate. Moreover, three amino acids (20 mol %) were utilized to generate iminium ions and subsequently to stabilize them by the presence of a carboxyl group. However, no conversion was observed after 7 days despite the additional structural elements as a carboxylic group in **AA-2** or guanidine motif in **AA-3** compared to simple **AA-1**.

**Scheme 3:**
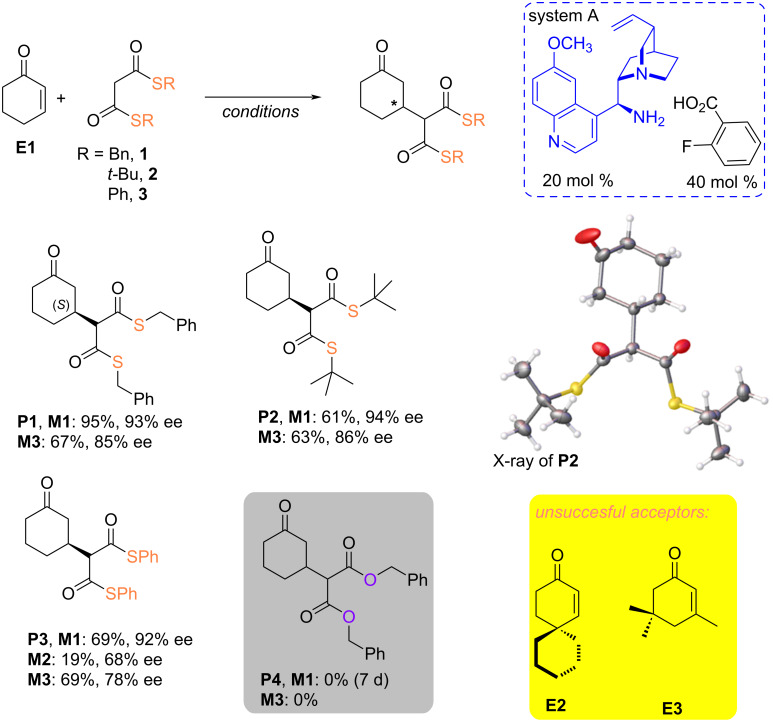
Impact of the bisthiomalonate on the yield and the stereoselectivity of the products.

Alterations in the thiol structure within thiomalonates significantly impact their stability and reactivity, thereby influencing both reaction efficiency and stereochemical outcome, as demonstrated in the case of catalysis employing bifunctional hydrogen bond donors [29]. In the context of imine catalysis using acid, the catalyst **AQ-1**'s ability to abstract protons from bisthiomalonate, thereby activating the nucleophile may be at least restricted. Consequently, we chose to investigate the impact of the thioester group on the outcome, including stereochemical, of the reaction between cyclohexenone and bisthiomalonates catalyzed by system A under standard conditions and mechanochemical (mixer mill) conditions (Scheme 3).

Under optimized conditions, the dibenzyl malonate adduct was formed in a ball mill with 67% yield and 85% ee of (*S*)-isomer, which was proved by X-ray studies. Assuming the mechanism of addition is similar in the case of other nucleophiles, the configuration of others was assigned by analogy. More sterically demanding di-*tert*-butyl thiomalonate (**2**) reacted in a similar fashion as the dibenzyl thiomalonate (**1**), leading also to desired products with 61% and 63% yield in the solution and mixer mill, respectively. However, the drop of stereoselectivity was noted in comparison to the reaction in toluene (94% ee vs 86% ee in mixer mill, ∆ee = 8). It should be noted that the dibenzyl malonate remained inactive for subjecting to reaction under both conditions. This result could be ascribed to the lower affinity of the oxo-ester to form active nucleophiles in comparison to the more favorable enolization of thiomalonates.

**Scheme 4:**
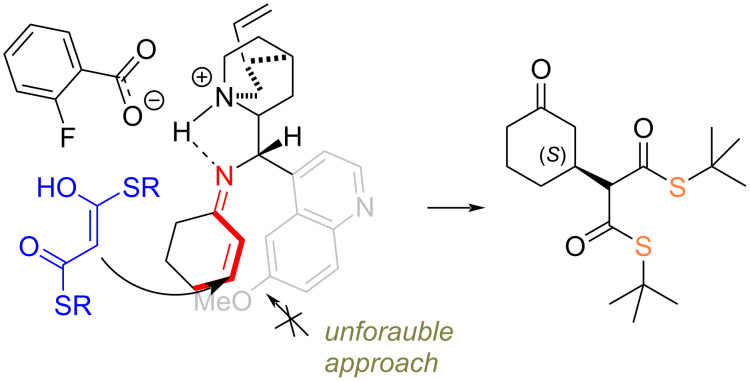
Plausible stereochemical model of the addition to cyclohexenone.

The stereochemical outcome of the thiomalonate’s addition could be rationalized by the assumption the iminium ion stabilized by an intramolecular hydrogen bond with a protonated amine unit activates the Michael acceptor (Scheme 4). Moreover, a strong but reversible covalent bond locates the electrophile upon the quinoline unit of the catalyst and thus subsequently blocks the bottom approach of the thiomalonate. Hence, such a sterically demanding nucleophile preferentially reaches the face of the reactive site from above the plane leading to the observed (*S*)-product.

The most pronounced effect exhibited by the thioester group concerning nucleophilic reactivity and stereochemical outcome of the reaction was observed for phenyl bis-thiomalonate **3**. The reaction in toluene required 24 hours to yield an enantiomerically enriched product (92% ee) with a 69% yield. However, achieving such a degree of over-reaction was associated with prolonged reaction times, which may affect the stability of both the substrate and its adduct. Hence, after 24 hours up to 9% of sulfa-adduct was formed (see Supporting Information File 1 for details). Unfavorable in this case is the formation of ketene via thiol elimination [40], leading to the formation of a mixture of products where the thia-Michael adduct predominates over the desired Michael product. We were sincerely interested in shortening the reaction time. This could be achieved by conducting the reaction under conditions of efficient mixing, increasing substrate concentration without solvents, and utilizing transient but repeatable pressure and temperature increases. These conditions are provided by mechanochemistry methods in ball mills. The reaction performed in a mixer mill over 1 hour only led to 13% of sulfa-Michael adduct (see Supporting Information File 1) beside the expected enantioenriched product (69%, 78% ee). We assumed the energy impact generated in the mixer mill could allow the reaction to occur also without the assistance of a catalyst, thereby following the non-stereoselective path. However, changing the milling system to a planetary ball mill affording fewer energy portions than the mixer mill, the Michael adduct was isolated with a rather low yield (19%) and moderate enantioselectivity (68%). Moreover, the steric bias in proximity (**E2**, Scheme 3) or the subunit of the conjugated system (**E3**) of acceptors revealed the nucleophiles (**1** and **3**) were unable to reach the reactive center or to form a stable bond.

**Scheme 5:**
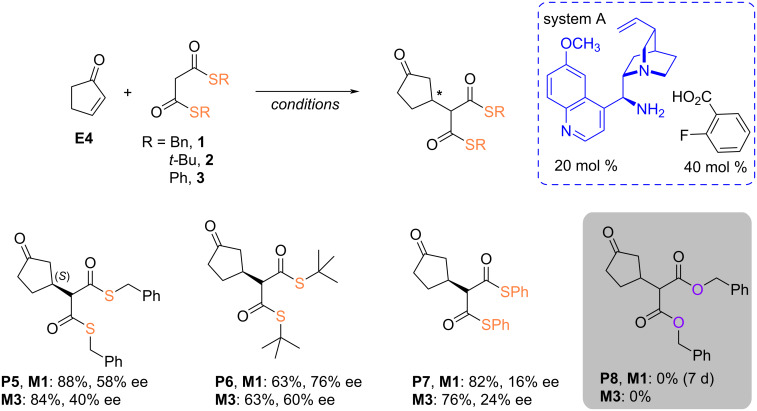
Addition of bisthiomalonates **1**–**3** to cyclopentenone.

Interestingly, when the reaction with bis-thiomalonate **3** was applied to cyclopentenone, which exhibits electrophilicity on the Mayer scale of −20.60 compared to cyclohexenone (*E* = −22.10, in DMSO) [41], comparable results were obtained both in solution and in a ball mill (Scheme 5). Therefore, a slightly more reactive electrophile does not significantly react less selectively in the ball mill than in solution, leading to the adduct with a low, 24% ee value, but higher than the analogous transformation in toluene. On the other hand, the transformation in the ball mill leads to the formation of 9% of the sulfa-Michael product (see Supporting Information File 1). However, the low stereoselectivity is not surprising. The analysis of the covalent adduct of cyclopentenone with amines may result from the “flattened” of the small system that renders them less sensitive to steric interactions generated by the catalyst compared to cyclohexenone [42–44]. In the case of thiomalonates **1** and **2**, reactions conducted in solution surpass those occurring in the ball mill in terms of stereoselectivity, and yield in case of **P5** and **P7**. Therefore, 1 hour in the ball mill versus 24 hours in solution is sufficient to obtain the desired product with similar efficiency.

**Scheme 6:**
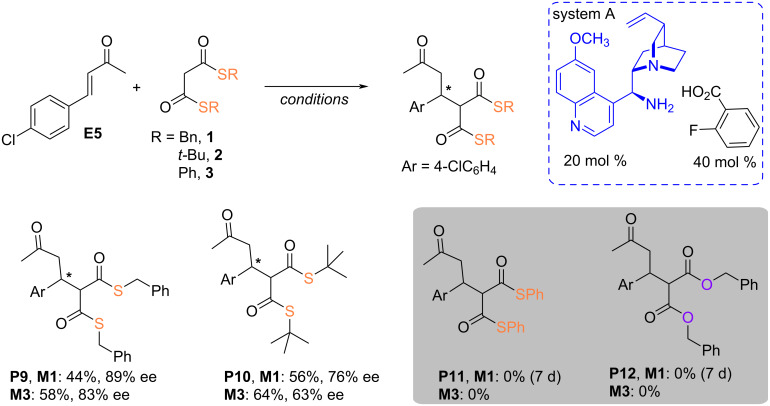
Acyclic enone in reactions with thiomalonates **1**–**4**.

Motivated by our curiosity surrounding the reactivity of alicyclic enones with bis-thiomalonates, we embarked on a study to evaluate a chosen catalytic system's efficacy under both standard conditions and mechanochemical milling. We aimed to facilitate the nucleophilic addition of **1**–**4** to 4-chlorobenzylideneacetone (**E5**). In contrast to previous examples, where the use of 1.5 equiv of the electrophile was a necessary step to ensure product formation in a ball mill (see Schemes 2–4), an equimolar mixture of acceptor **E5** and nucleophile **1**–**4** was subjected to Michael addition reaction using system A (Scheme 6). Despite the long reaction time (45 h for thiomalonate **1** up to 70 h for nucleophile **2**), the expected adducts were obtained with yields of 44% and 56%, respectively. Similar transformations conducted in a ball mill afforded products with slightly higher yields (58% for **1** and 64% for **2**) and slightly lower stereoselectivities requiring only 1 hour instead of 45 and 70 hours. Unfortunately, attempts to add thiomalonate **3** and dibenzyl malonate failed. In the first case, instead of forming the expected product, only the thiophenol adduct is formed (see Scheme 2, right chart, for the structure of sulfa-Michael adduct; R = Ph).

**Scheme 7:**
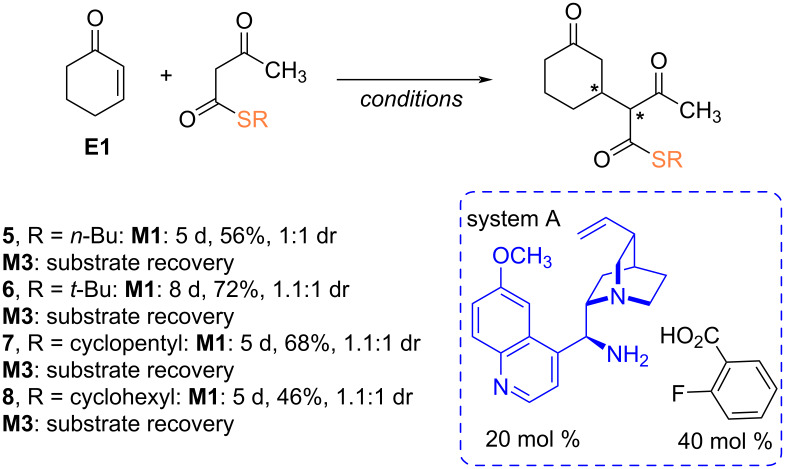
Reaction of β-ketothioesters with acceptor **E1**.

Finally, an interesting group of β-ketothioesters **5**–**8**, which are rarely utilized in asymmetric catalysis [45], were employed as nucleophiles in reactions with acceptor **E1** (Scheme 7). While additions of various β-ketothioesters, including **5**–**8**, to nitrostyrenes proceeded efficiently, leading to products with enantiomeric excesses above 95% ee [46], changing the catalytic system and activation method from non-covalent to covalent substrate activation, allowed for the synthesis of products with moderate to good yields (46–72%). However, it was not possible to determine the enantiomeric excesses for nearly equimolar pairs of diastereomeric products due to the lack of appropriate conditions for resolution among the six tested chiral stationary phases. To our surprise, reactions conducted in the ball mill did not lead to the formation of new C–C bonds, and even after extending the reaction time, a mixture of unreacted reagents was recovered.

The observations indicate a significant role of factors related to the physical contact between substances, milling balls, and the walls of the grinding vessel on the final outcome of the reaction when the system is in a liquid state. Interestingly, the transformations conducted under ball mill conditions only failed when the crude product, rather than the initial system, was in a liquid state. Thus, while the reaction of liquid enones with thiomalonate **2** successfully yielded the expected products, the adducts **5**–**8** were only formed in solution when they were in a liquid form. We assume the keto–enol interactions between the substrate molecules or substrate reagents might essentially decreased the reactivity in comparison to solution, as indicated in the recent precedent [47].

## Conclusion

Mechanochemistry, particularly ball milling, represents a promising avenue for unconventional reaction pathways by exploiting kinetic energy to surpass equilibrium constraints. This methodology presents several advantages over conventional solvent-based approaches, including the potential for diverse product formation with limited substrates and the development of sustainable transformations characterized by reduced reaction times.

An unprecedented combination of mechanochemistry with organocatalysis, notably chiral amine-catalyzed stereoselective reactions, has been extensively investigated. While primary amine-catalyzed reactions under ball milling conditions are less documented, they offer prospects for substrate diversification using a more sustainable approach. Efforts to enhance reactivity through nucleophile modifications, such as transitioning from oxoesters to thioesters, show promise in enhancing reaction progress. However, the efficacy of catalysts varies, with certain systems proving ineffective or leading to diminished stereoselectivity under mechanochemical conditions. Despite a slight decrease in stereoselectivity with the usual ∆ee of about 8%, reactions conducted in the mill allow for reaction completion within 1 hour compared to the 24 to 168 hours needed when conducting reactions in solution. Therefore, thiomalonates exhibit distinctive reactivity compared to oxoesters, impacting reaction efficiency. Moreover, despite being more reactive acylating agents than oxoesters, thioesters are effectively utilized as nucleophiles in reactions catalyzed by primary amines in transformation requiring the formation of the imines without catalyst deactivation. The study underscores the potential of mechanochemistry in broadening the scope of organocatalytic and stereoselective transformations including the application of rather unexplored thioesters.

## Supporting Information

File 1 Experimental and analytical data, crystallographic information and NMR spectra.

## Data Availability

All data that supports the findings of this study is available in the published article and/or the supporting information to this article.

## References

[1] Margetić D (2023). Pure Appl Chem.

[2] Cuccu F, De Luca L, Delogu F, Colacino E, Solin N, Mocci R, Porcheddu A (2022). ChemSusChem.

[3] Howard J L, Cao Q, Browne D L (2018). Chem Sci.

[4] Hernández J G, Bolm C (2017). J Org Chem.

[5] James S L, Adams C J, Bolm C, Braga D, Collier P, Friščić T, Grepioni F, Harris K D M, Hyett G, Jones W (2012). Chem Soc Rev.

[6] Pladevall B S, de Aguirre A, Maseras F (2021). ChemSusChem.

[7] Delogu F, Takács L (2018). J Mater Sci.

[8] Chen Z, Lu S, Mao Q, Buekens A, Wang Y, Yan J (2017). Environ Sci Pollut Res.

[9] Némethová V, Krištofíková D, Mečiarová M, Šebesta R (2023). Chem Rec.

[10] Juaristi E, Avila-Ortiz C G (2023). Synthesis.

[11] Chauhan P, Chimni S S (2012). Beilstein J Org Chem.

[12] Rodríguez B, Rantanen T, Bolm C (2006). Angew Chem, Int Ed.

[13] Rodríguez B, Bruckmann A, Bolm C (2007). Chem – Eur J.

[14] Hernández J G, Juaristi E (2011). J Org Chem.

[15] Zhang Y, Duan H-X, Wang Y-Q (2020). Chin J Org Chem.

[16] Melchiorre P (2012). Angew Chem, Int Ed.

[17] Brazier J B, Tomkinson N C O (2010). Top Curr Chem.

[18] Krištofíková D, Modrocká V, Mečiarová M, Šebesta R (2020). ChemSusChem.

[19] Procopio A, De Nino A, Nardi M, Oliverio M, Paonessa R, Pasceri R (2010). Synlett.

[20] Mao Z, Jia Y, Li W, Wang R (2010). J Org Chem.

[21] Li P, Wen S, Yu F, Liu Q, Li W, Wang Y, Liang X, Ye J (2009). Org Lett.

[22] Dudziński K, Pakulska A M, Kwiatkowski P (2012). Org Lett.

[23] Miyamae N, Watanabe N, Moritaka M, Nakano K, Ichikawa Y, Kotsuki H (2014). Org Biomol Chem.

[24] Luo C, Jin Y, Du D-M (2012). Org Biomol Chem.

[25] Kamito Y, Masuda A, Yuasa H, Tada N, Itoh A, Nakashima K, Hirashima S-i, Koseki Y, Miura T (2014). Tetrahedron: Asymmetry.

[26] Castro E A (1999). Chem Rev.

[27] Hupe D J, Jencks W P (1977). J Am Chem Soc.

[28] Pan Y, Kee C W, Jiang Z, Ma T, Zhao Y, Yang Y, Xue H, Tan C-H (2011). Chem – Eur J.

[29] Mała Ż A, Janicki M J, Góra R W, Konieczny K A, Kowalczyk R (2023). Adv Synth Catal.

[30] Liu H-J, Oppong I V (1982). Can J Chem.

[31] Ye W, Jiang Z, Zhao Y, Goh S L M, Leow D, Soh Y-T, Tan C-H (2007). Adv Synth Catal.

[32] Van Eldik R, Asano T, Le Noble W J (1989). Chem Rev.

[33] Drljaca A, Hubbard C D, van Eldik R, Asano T, Basilevsky M V, le Noble W J (1998). Chem Rev.

[34] Schettino V, Bini R (2007). Chem Soc Rev.

[35] Kwiatkowski P, Dudziński K, Łyżwa D (2011). Org Lett.

[36] Dalpozzo R, Bartoli G, Bencivenni G (2011). Symmetry.

[37] Song C E, Park S J, Hwang I-S, Jung M J, Shim S Y, Bae H Y, Jung J Y (2019). Nat Commun.

[38] Ahrendt K A, Borths C J, MacMillan D W C (2000). J Am Chem Soc.

[39] Kowalczyk R, Boratyński P J, Wierzba A J, Bąkowicz J (2015). RSC Adv.

[40] Yang W, Drueckhammer D G (2001). J Am Chem Soc.

[41] Mayer R J, Allihn P W A, Hampel N, Mayer P, Sieber S A, Ofial A R (2021). Chem Sci.

[42] Sanders J N, Jun H, Yu R A, Gleason J L, Houk K N (2020). J Am Chem Soc.

[43] Wang X, Reisinger C M, List B (2008). J Am Chem Soc.

[44] Lifchits O, Mahlau M, Reisinger C M, Lee A, Farès C, Polyak I, Gopakumar G, Thiel W, List B (2013). J Am Chem Soc.

[45] Wang X, Ji Z, Liu J, Wang B, Jin H, Zhang L (2023). Acta Chim Sin (Chin Ed).

[46] Dajek M, Kubiak P, Bąkowicz J, Dziuk B, Kowalczyk R (2024). ChemRxiv.

[47] Kralj M, Lukin S, Miletić G, Halasz I (2021). J Org Chem.

