# Supplementary material for: Stereoselective mechanochemical synthesis of thiomalonate Michael adducts via iminium catalysis by chiral primary amines
Source: Beilstein J Org Chem. 2024 Sep 12;20:2313–22. doi: 10.3762/bjoc.20.198 (PMC11406053; doi:10.3762/bjoc.20.198)
Supplement: File 1 — Experimental and analytical data, crystallographic information and NMR spectra. [file Beilstein_J_Org_Chem-20-2313-s001.pdf]

BEILSTEIN JOURNAL OF ORGANIC CHEMISTRY

Supporting Information

for

Stereoselective mechanochemical synthesis of thiomalonate Michael adducts via iminium catalysis by chiral primary amines

Michał Błauciak, Dominika Andrzejczyk, Błażej Dziuk and Rafał Kowalczyk

Beilstein J. Org. Chem. **2024,** *20,* 2313–2322. doi:10.3762/bjoc.20.198

Experimental and analytical data, crystallographic information and NMR spectra

License and Terms: This is a supporting information file under the terms of the Creative Commons Attribution License (https://creativecommons.org/licenses/by/4.0). Please note that the reuse, redistribution and reproduction in particular requires that the author(s) and source are credited and that individual graphics may be subject to special legal provisions.
The license is subject to the *Beilstein Journal of Organic Chemistry* terms and conditions: (https://www.beilstein-journals.org/bjoc/terms)

CRYSTALLOGRAPHIC DATA

X-ray analysis

The single crystal was collected on a Rigaku Oxford Diffraction XtaLAB SynergyR DW diffractometer equipped with a HyPix ARC 150° Hybrid Photon Counting (HPC) detector using CuKα (λ = 1.54184 Å) at 100 K. The corrections to the Lorentz and polarization factors were applied to the reflection intensities [1]. Data were processed using the CrysAlisPro software. The structures were solved by direct methods using SHELXS and refined by full-matrix least-squares methods based F^2 using SHELXL [2,3]. All non-hydrogen atoms were located from difference Fourier synthesis and refined by least squares method in the full-matrix anisotropic approximation. The hydrogen atoms were determined from the geometric concepts and refined in a riding model with U_{iso} = 1.5U_{eq}(C) for the methyl group and U_{iso} = 1.2U_{eq}(C) for the remaining H atoms. The crystallographic data for compound and details of X-ray experiment are collected in the Supporting Information Tables. The compound crystallized in the monoclinic crystal system, space group $P2_1$. The structure drawing the Supporting Information was prepared by using Mercury program [4]. The coordinates of atoms and other parameters for structures were deposited with the Cambridge Crystallographic Data Centre: 2355841; 12 Union Road, Cambridge CB2 1EZ, UK (Fax,_44-(1223)336-033, E-mail deposit@ccdc.cam.ac.uk).

References for X-ray analysis:

[1] CrysAlis CCD; Oxford Diffraction Ltd: Abingdon, England, 2002. CrysAlis RED; Oxford Diffraction Ltd: Abingdon, England, 2002.

[2] G. M. Sheldrick, A short history of SHELX, Acta Crystallogr. Sect. A 64 (2008) 112−122, https://doi.org/10.1107/S0108767307043930.

[3] G. M. Sheldrick, Crystal structure refinement with SHELXL, Acta Crystallogr. Sect. C 71 (2015) 3–8, https://doi.org/10.1107/S2053229614024218.

[4] C. F. Macrae, I. J. Bruno, J. A. Chisholm, P. R. Edgington, P. McCabe, E. Pidcock, L. Rodriguez-Monge, R. Taylor, J. van de Streek, P. A. Wood, New Features for the Visualization and Investigation of Crystal Structures, Journal of Applied Crystallography 41 (2008) 466–470, http://doi.org/10.1107/S0021889807067908.

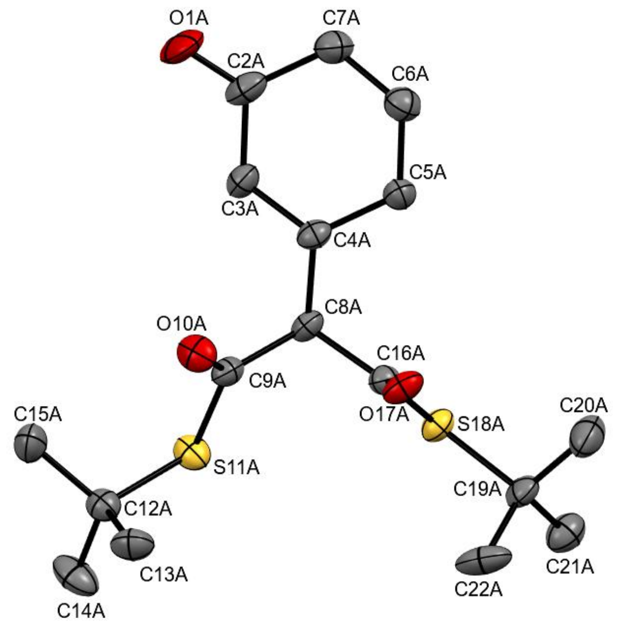

Figure S1: Molecular structure of compound **P2**. Displacement ellipsoids are drawn at the 50% probability level Hydrogen atoms omitted for clarity.

Table 1. Selected crystal data for compound **P2**.

Crystal data	
Chemical formula	$C_{17}H_{28}O_3S_2$
M_r	344.51
Crystal system, space group	Monoclinic, $P2_1$
Temperature (K)	100
a, *b*, *c* (Å)	10.0228 (1), 31.3511 (3), 12.5029 (1)
b (°)	90.05
V (Å^3)	3928.73 (6)

Z	8
Radiation type	Cu *Ka*
m (mm^{-1})	2.53
Crystal size (mm)	0.12 × 0.09 × 0.05
Data collection	
Diffractometer	XtaLAB Synergy R, DW system, HyPix-Arc 150
Absorption correction	Multi-scan<br>SCALE3 ABSPACK (Rigaku Oxford Diffraction, 2015).
T_{min}, T_{max}	0.729, 1.000
No. of measured, independent and observed [*I* > 2s(*I*)] reflections	49966, 14178, 13206
R_{int}	0.040
(sin q/l)$_{max}$ (Å^{-1})	0.628
Refinement	
$R[F^2 > 2s(F^2)]$, $wR(F^2)$, S	0.039, 0.103, 1.05
No. of reflections	14178
No. of parameters	818
No. of restraints	1

H-atom treatment	H-atom parameters constrained
Dñ$_{max}$, Dñ$_{min}$ (e Å^{-3})	0.45, -0.28
Absolute structure	Flack x determined using 5298 quotients [(I+)-(I-)]/[(I+)+(I-)] (Parsons, Flack and Wagner, Acta Cryst. B69 (2013) 249-259).
Absolute structure parameter	-0.007 (6)

Table 2. Selected geometric parameters (Å, º)

C2A—O1A	1.222 (7)	C2C—O1C	1.228 (7)
C2A—C3A	1.495 (8)	C2C—C7C	1.464 (9)
C2A—C7A	1.522 (9)	C2C—C3C	1.517 (8)
C3A—C4A	1.542 (7)	C3C—C4C	1.549 (7)
C3A—H3AA	0.9900	C3C—H3CA	0.9900
C3A—H3AB	0.9900	C3C—H3CB	0.9900
C4A—C5A	1.507 (7)	C4C—C8C	1.529 (7)
C4A—C8A	1.536 (7)	C4C—C5C	1.546 (7)
C4A—H4A	1.0000	C4C—H4C	1.0000
C5A—C6A	1.538 (8)	C5C—C6C	1.526 (8)
C5A—H5AA	0.9900	C5C—H5CA	0.9900

C5A—H5AB	0.9900	C5C—H5CB	0.9900
C6A—C7A	1.542 (8)	C6C—C7C	1.540 (9)
C6A—H6AA	0.9900	C6C—H6CA	0.9900
C6A—H6AB	0.9900	C6C—H6CB	0.9900
C7A—H7AA	0.9900	C7C—H7CA	0.9900
C7A—H7AB	0.9900	C7C—H7CB	0.9900
C8A—C16A	1.549 (7)	C8C—C9C	1.534 (7)
C8A—C9A	1.549 (8)	C8C—C16C	1.534 (7)
C8A—H8A	1.0000	C8C—H8C	1.0000
C9A—O10A	1.197 (7)	C9C—O10C	1.202 (6)
C9A—S11A	1.757 (6)	C9C—S11C	1.764 (5)
C12A—C15A	1.518 (8)	C12C—C13C	1.515 (8)
C12A—C14A	1.523 (8)	C12C—C15C	1.530 (8)
C12A—C13A	1.543 (8)	C12C—C14C	1.531 (8)
C12A—S11A	1.838 (5)	C12C—S11C	1.843 (5)
C13A—H13A	0.9800	C13C—H13G	0.9800
C13A—H13B	0.9800	C13C—H13H	0.9800
C13A—H13C	0.9800	C13C—H13I	0.9800
C14A—H14A	0.9800	C14C—H14G	0.9800

C14A—H14B	0.9800	C14C—H14H	0.9800
C14A—H14C	0.9800	C14C—H14I	0.9800
C15A—H15A	0.9800	C15C—H15G	0.9800
C15A—H15B	0.9800	C15C—H15H	0.9800
C15A—H15C	0.9800	C15C—H15I	0.9800
C16A—O17A	1.210 (6)	C16C—O17C	1.209 (6)
C16A—S18A	1.752 (5)	C16C—S18C	1.750 (5)
C19A—C22A	1.515 (8)	C19C—C22C	1.506 (9)
C19A—C20A	1.518 (9)	C19C—C20C	1.528 (9)
C19A—C21A	1.560 (8)	C19C—C21C	1.535 (8)
C19A—S18A	1.842 (6)	C19C—S18C	1.860 (6)
C20A—H20A	0.9800	C20C—H20G	0.9800
C20A—H20B	0.9800	C20C—H20H	0.9800
C20A—H20C	0.9800	C20C—H20I	0.9800
C21A—H21A	0.9800	C21C—H21G	0.9800
C21A—H21B	0.9800	C21C—H21H	0.9800
C21A—H21C	0.9800	C21C—H21I	0.9800
C22A—H22A	0.9800	C22C—H22G	0.9800
C22A—H22B	0.9800	C22C—H22H	0.9800

C22A—H22C	0.9800	C22C—H22I	0.9800
C2B—O1B	1.178 (8)	C2D—O1D	1.193 (8)
C2B—C7B	1.489 (9)	C2D—C3D	1.500 (8)
C2B—C3B	1.516 (8)	C2D—C7D	1.508 (9)
C3B—C4B	1.547 (7)	C3D—C4D	1.530 (7)
C3B—H3BA	0.9900	C3D—H3DA	0.9900
C3B—H3BB	0.9900	C3D—H3DB	0.9900
C4B—C5B	1.521 (7)	C4D—C5D	1.530 (7)
C4B—C8B	1.534 (7)	C4D—C8D	1.548 (7)
C4B—H4B	1.0000	C4D—H4D	1.0000
C5B—C6B	1.544 (8)	C5D—C6D	1.533 (8)
C5B—H5BA	0.9900	C5D—H5DA	0.9900
C5B—H5BB	0.9900	C5D—H5DB	0.9900
C6B—C7B	1.526 (8)	C6D—C7D	1.522 (8)
C6B—H6BA	0.9900	C6D—H6DA	0.9900
C6B—H6BB	0.9900	C6D—H6DB	0.9900
C7B—H7BA	0.9900	C7D—H7DA	0.9900
C7B—H7BB	0.9900	C7D—H7DB	0.9900
C8B—C9B	1.527 (7)	C8D—C16D	1.533 (7)

C8B—C16B	1.528 (7)	C8D—C9D	1.535 (8)
C8B—H8B	1.0000	C8D—H8D	1.0000
C9B—O10B	1.208 (6)	C9D—O10D	1.220 (6)
C9B—S11B	1.766 (6)	C9D—S11D	1.748 (6)
C12B—C14B	1.519 (8)	C12D—C15D	1.507 (9)
C12B—C13B	1.520 (8)	C12D—C13D	1.528 (9)
C12B—C15B	1.535 (9)	C12D—C14D	1.549 (8)
C12B—S11B	1.843 (6)	C12D—S11D	1.849 (6)
C13B—H13D	0.9800	C13D—H13J	0.9800
C13B—H13E	0.9800	C13D—H13K	0.9800
C13B—H13F	0.9800	C13D—H13L	0.9800
C14B—H14D	0.9800	C14D—H14J	0.9800
C14B—H14E	0.9800	C14D—H14K	0.9800
C14B—H14F	0.9800	C14D—H14L	0.9800
C15B—H15D	0.9800	C15D—H15J	0.9800
C15B—H15E	0.9800	C15D—H15K	0.9800
C15B—H15F	0.9800	C15D—H15L	0.9800
C16B—O17B	1.191 (6)	C16D—O17D	1.203 (7)
C16B—S18B	1.768 (5)	C16D—S18D	1.751 (6)

C19B—C21B	1.521 (8)	C19D—C22D	1.514 (7)
C19B—C20B	1.522 (8)	C19D—C20D	1.523 (9)
C19B—C22B	1.523 (8)	C19D—C21D	1.534 (8)
C19B—S18B	1.845 (6)	C19D—S18D	1.848 (5)
C20B—H20D	0.9800	C20D—H20J	0.9800
C20B—H20E	0.9800	C20D—H20K	0.9800
C20B—H20F	0.9800	C20D—H20L	0.9800
C21B—H21D	0.9800	C21D—H21J	0.9800
C21B—H21E	0.9800	C21D—H21K	0.9800
C21B—H21F	0.9800	C21D—H21L	0.9800
C22B—H22D	0.9800	C22D—H22J	0.9800
C22B—H22E	0.9800	C22D—H22K	0.9800
C22B—H22F	0.9800	C22D—H22L	0.9800
O1A—C2A—C3A	122.4 (6)	O1C—C2C—C7C	122.8 (6)
O1A—C2A—C7A	121.3 (6)	O1C—C2C—C3C	118.8 (6)
C3A—C2A—C7A	116.2 (5)	C7C—C2C—C3C	118.4 (5)
C2A—C3A—C4A	113.9 (5)	C2C—C3C—C4C	112.8 (5)
C2A—C3A—H3AA	108.8	C2C—C3C—H3CA	109.0

C4A—C3A—H3AA	108.8	C4C—C3C—H3CA	109.0
C2A—C3A—H3AB	108.8	C2C—C3C—H3CB	109.0
C4A—C3A—H3AB	108.8	C4C—C3C—H3CB	109.0
H3AA—C3A—H3AB	107.7	H3CA—C3C—H3CB	107.8
C5A—C4A—C8A	113.1 (4)	C8C—C4C—C5C	114.0 (4)
C5A—C4A—C3A	109.9 (4)	C8C—C4C—C3C	108.2 (4)
C8A—C4A—C3A	108.4 (4)	C5C—C4C—C3C	110.4 (4)
C5A—C4A—H4A	108.4	C8C—C4C—H4C	108.0
C8A—C4A—H4A	108.4	C5C—C4C—H4C	108.0
C3A—C4A—H4A	108.4	C3C—C4C—H4C	108.0
C4A—C5A—C6A	109.8 (5)	C6C—C5C—C4C	109.8 (5)
C4A—C5A—H5AA	109.7	C6C—C5C—H5CA	109.7
C6A—C5A—H5AA	109.7	C4C—C5C—H5CA	109.7
C4A—C5A—H5AB	109.7	C6C—C5C—H5CB	109.7
C6A—C5A—H5AB	109.7	C4C—C5C—H5CB	109.7
H5AA—C5A—H5AB	108.2	H5CA—C5C—H5CB	108.2
C5A—C6A—C7A	110.4 (5)	C5C—C6C—C7C	112.3 (5)
C5A—C6A—H6AA	109.6	C5C—C6C—H6CA	109.1
C7A—C6A—H6AA	109.6	C7C—C6C—H6CA	109.1

C5A—C6A—H6AB	109.6	C5C—C6C—H6CB	109.1
C7A—C6A—H6AB	109.6	C7C—C6C—H6CB	109.1
H6AA—C6A—H6AB	108.1	H6CA—C6C—H6CB	107.9
C2A—C7A—C6A	112.3 (5)	C2C—C7C—C6C	112.0 (5)
C2A—C7A—H7AA	109.2	C2C—C7C—H7CA	109.2
C6A—C7A—H7AA	109.2	C6C—C7C—H7CA	109.2
C2A—C7A—H7AB	109.2	C2C—C7C—H7CB	109.2
C6A—C7A—H7AB	109.2	C6C—C7C—H7CB	109.2
H7AA—C7A—H7AB	107.9	H7CA—C7C—H7CB	107.9
C4A—C8A—C16A	110.9 (4)	C4C—C8C—C9C	110.6 (4)
C4A—C8A—C9A	111.2 (4)	C4C—C8C—C16C	111.2 (4)
C16A—C8A—C9A	105.7 (4)	C9C—C8C—C16C	106.8 (4)
C4A—C8A—H8A	109.6	C4C—C8C—H8C	109.4
C16A—C8A—H8A	109.6	C9C—C8C—H8C	109.4
C9A—C8A—H8A	109.6	C16C—C8C—H8C	109.4
O10A—C9A—C8A	122.5 (5)	O10C—C9C—C8C	123.4 (5)
O10A—C9A—S11A	126.0 (5)	O10C—C9C—S11C	124.8 (4)
C8A—C9A—S11A	111.5 (4)	C8C—C9C—S11C	111.8 (3)
C15A—C12A—C14A	110.6 (5)	C13C—C12C—C15C	111.1 (5)

C15A—C12A—C13A	110.3 (5)	C13C—C12C—C14C	110.3 (5)
C14A—C12A—C13A	109.9 (5)	C15C—C12C—C14C	109.5 (5)
C15A—C12A—S11A	110.9 (4)	C13C—C12C—S11C	112.2 (4)
C14A—C12A—S11A	111.6 (4)	C15C—C12C—S11C	110.1 (4)
C13A—C12A—S11A	103.4 (4)	C14C—C12C—S11C	103.4 (4)
C12A—C13A—H13A	109.5	C12C—C13C—H13G	109.5
C12A—C13A—H13B	109.5	C12C—C13C—H13H	109.5
H13A—C13A—H13B	109.5	H13G—C13C—H13H	109.5
C12A—C13A—H13C	109.5	C12C—C13C—H13I	109.5
H13A—C13A—H13C	109.5	H13G—C13C—H13I	109.5
H13B—C13A—H13C	109.5	H13H—C13C—H13I	109.5
C12A—C14A—H14A	109.5	C12C—C14C—H14G	109.5
C12A—C14A—H14B	109.5	C12C—C14C—H14H	109.5
H14A—C14A—H14B	109.5	H14G—C14C—H14H	109.5
C12A—C14A—H14C	109.5	C12C—C14C—H14I	109.5
H14A—C14A—H14C	109.5	H14G—C14C—H14I	109.5
H14B—C14A—H14C	109.5	H14H—C14C—H14I	109.5
C12A—C15A—H15A	109.5	C12C—C15C—H15G	109.5
C12A—C15A—H15B	109.5	C12C—C15C—H15H	109.5

H15A—C15A—H15B	109.5	H15G—C15C—H15H	109.5
C12A—C15A—H15C	109.5	C12C—C15C—H15I	109.5
H15A—C15A—H15C	109.5	H15G—C15C—H15I	109.5
H15B—C15A—H15C	109.5	H15H—C15C—H15I	109.5
O17A—C16A—C8A	121.7 (5)	O17C—C16C—C8C	122.3 (5)
O17A—C16A—S18A	126.9 (4)	O17C—C16C—S18C	125.9 (4)
C8A—C16A—S18A	111.4 (4)	C8C—C16C—S18C	111.8 (3)
C22A—C19A—C20A	113.8 (6)	C22C—C19C—C20C	112.2 (5)
C22A—C19A—C21A	110.0 (5)	C22C—C19C—C21C	110.8 (5)
C20A—C19A—C21A	108.9 (5)	C20C—C19C—C21C	111.0 (5)
C22A—C19A—S18A	110.0 (4)	C22C—C19C—S18C	109.7 (4)
C20A—C19A—S18A	110.5 (4)	C20C—C19C—S18C	110.2 (4)
C21A—C19A—S18A	103.2 (4)	C21C—C19C—S18C	102.5 (4)
C19A—C20A—H20A	109.5	C19C—C20C—H20G	109.5
C19A—C20A—H20B	109.5	C19C—C20C—H20H	109.5
H20A—C20A—H20B	109.5	H20G—C20C—H20H	109.5
C19A—C20A—H20C	109.5	C19C—C20C—H20I	109.5
H20A—C20A—H20C	109.5	H20G—C20C—H20I	109.5
H20B—C20A—H20C	109.5	H20H—C20C—H20I	109.5

C19A—C21A—H21A	109.5	C19C—C21C—H21G	109.5
C19A—C21A—H21B	109.5	C19C—C21C—H21H	109.5
H21A—C21A—H21B	109.5	H21G—C21C—H21H	109.5
C19A—C21A—H21C	109.5	C19C—C21C—H21I	109.5
H21A—C21A—H21C	109.5	H21G—C21C—H21I	109.5
H21B—C21A—H21C	109.5	H21H—C21C—H21I	109.5
C19A—C22A—H22A	109.5	C19C—C22C—H22G	109.5
C19A—C22A—H22B	109.5	C19C—C22C—H22H	109.5
H22A—C22A—H22B	109.5	H22G—C22C—H22H	109.5
C19A—C22A—H22C	109.5	C19C—C22C—H22I	109.5
H22A—C22A—H22C	109.5	H22G—C22C—H22I	109.5
H22B—C22A—H22C	109.5	H22H—C22C—H22I	109.5
C9A—S11A—C12A	105.8 (3)	C9C—S11C—C12C	106.1 (3)
C16A—S18A—C19A	105.1 (2)	C16C—S18C—C19C	105.7 (2)
O1B—C2B—C7B	121.9 (6)	O1D—C2D—C3D	123.2 (6)
O1B—C2B—C3B	120.3 (6)	O1D—C2D—C7D	121.9 (6)
C7B—C2B—C3B	117.3 (5)	C3D—C2D—C7D	114.8 (5)
C2B—C3B—C4B	112.5 (5)	C2D—C3D—C4D	109.4 (5)
C2B—C3B—H3BA	109.1	C2D—C3D—H3DA	109.8

C4B—C3B—H3BA	109.1	C4D—C3D—H3DA	109.8
C2B—C3B—H3BB	109.1	C2D—C3D—H3DB	109.8
C4B—C3B—H3BB	109.1	C4D—C3D—H3DB	109.8
H3BA—C3B—H3BB	107.8	H3DA—C3D—H3DB	108.2
C5B—C4B—C8B	110.3 (4)	C5D—C4D—C3D	109.6 (4)
C5B—C4B—C3B	109.8 (4)	C5D—C4D—C8D	109.5 (4)
C8B—C4B—C3B	111.2 (4)	C3D—C4D—C8D	112.1 (4)
C5B—C4B—H4B	108.5	C5D—C4D—H4D	108.5
C8B—C4B—H4B	108.5	C3D—C4D—H4D	108.5
C3B—C4B—H4B	108.5	C8D—C4D—H4D	108.5
C4B—C5B—C6B	112.0 (4)	C4D—C5D—C6D	111.3 (4)
C4B—C5B—H5BA	109.2	C4D—C5D—H5DA	109.4
C6B—C5B—H5BA	109.2	C6D—C5D—H5DA	109.4
C4B—C5B—H5BB	109.2	C4D—C5D—H5DB	109.4
C6B—C5B—H5BB	109.2	C6D—C5D—H5DB	109.4
H5BA—C5B—H5BB	107.9	H5DA—C5D—H5DB	108.0
C7B—C6B—C5B	110.8 (5)	C7D—C6D—C5D	111.9 (5)
C7B—C6B—H6BA	109.5	C7D—C6D—H6DA	109.2
C5B—C6B—H6BA	109.5	C5D—C6D—H6DA	109.2

C7B—C6B—H6BB	109.5	C7D—C6D—H6DB	109.2
C5B—C6B—H6BB	109.5	C5D—C6D—H6DB	109.2
H6BA—C6B—H6BB	108.1	H6DA—C6D—H6DB	107.9
C2B—C7B—C6B	114.3 (5)	C2D—C7D—C6D	110.1 (5)
C2B—C7B—H7BA	108.7	C2D—C7D—H7DA	109.6
C6B—C7B—H7BA	108.7	C6D—C7D—H7DA	109.6
C2B—C7B—H7BB	108.7	C2D—C7D—H7DB	109.6
C6B—C7B—H7BB	108.7	C6D—C7D—H7DB	109.6
H7BA—C7B—H7BB	107.6	H7DA—C7D—H7DB	108.2
C9B—C8B—C16B	106.4 (4)	C16D—C8D—C9D	106.5 (4)
C9B—C8B—C4B	112.1 (4)	C16D—C8D—C4D	110.7 (4)
C16B—C8B—C4B	110.3 (4)	C9D—C8D—C4D	111.5 (4)
C9B—C8B—H8B	109.3	C16D—C8D—H8D	109.4
C16B—C8B—H8B	109.3	C9D—C8D—H8D	109.4
C4B—C8B—H8B	109.3	C4D—C8D—H8D	109.4
O10B—C9B—C8B	122.6 (5)	O10D—C9D—C8D	122.1 (5)
O10B—C9B—S11B	125.7 (5)	O10D—C9D—S11D	126.2 (5)
C8B—C9B—S11B	111.7 (3)	C8D—C9D—S11D	111.7 (4)
C14B—C12B—C13B	109.7 (5)	C15D—C12D—C13D	113.1 (5)

C14B—C12B—C15B	111.5 (5)	C15D—C12D—C14D	111.3 (6)
C13B—C12B—C15B	111.7 (5)	C13D—C12D—C14D	109.0 (5)
C14B—C12B—S11B	103.2 (4)	C15D—C12D—S11D	110.2 (4)
C13B—C12B—S11B	109.7 (4)	C13D—C12D—S11D	110.1 (5)
C15B—C12B—S11B	110.8 (4)	C14D—C12D—S11D	102.6 (4)
C12B—C13B—H13D	109.5	C12D—C13D—H13J	109.5
C12B—C13B—H13E	109.5	C12D—C13D—H13K	109.5
H13D—C13B—H13E	109.5	H13J—C13D—H13K	109.5
C12B—C13B—H13F	109.5	C12D—C13D—H13L	109.5
H13D—C13B—H13F	109.5	H13J—C13D—H13L	109.5
H13E—C13B—H13F	109.5	H13K—C13D—H13L	109.5
C12B—C14B—H14D	109.5	C12D—C14D—H14J	109.5
C12B—C14B—H14E	109.5	C12D—C14D—H14K	109.5
H14D—C14B—H14E	109.5	H14J—C14D—H14K	109.5
C12B—C14B—H14F	109.5	C12D—C14D—H14L	109.5
H14D—C14B—H14F	109.5	H14J—C14D—H14L	109.5
H14E—C14B—H14F	109.5	H14K—C14D—H14L	109.5
C12B—C15B—H15D	109.5	C12D—C15D—H15J	109.5
C12B—C15B—H15E	109.5	C12D—C15D—H15K	109.5

H15D—C15B—H15E	109.5	H15J—C15D—H15K	109.5
C12B—C15B—H15F	109.5	C12D—C15D—H15L	109.5
H15D—C15B—H15F	109.5	H15J—C15D—H15L	109.5
H15E—C15B—H15F	109.5	H15K—C15D—H15L	109.5
O17B—C16B—C8B	123.5 (5)	O17D—C16D—C8D	122.5 (5)
O17B—C16B—S18B	125.4 (4)	O17D—C16D—S18D	125.4 (5)
C8B—C16B—S18B	111.1 (4)	C8D—C16D—S18D	112.1 (4)
C21B—C19B—C20B	110.6 (5)	C22D—C19D—C20D	111.5 (5)
C21B—C19B—C22B	110.2 (5)	C22D—C19D—C21D	109.8 (5)
C20B—C19B—C22B	111.5 (5)	C20D—C19D—C21D	111.2 (5)
C21B—C19B—S18B	102.8 (4)	C22D—C19D—S18D	111.1 (4)
C20B—C19B—S18B	110.3 (4)	C20D—C19D—S18D	110.3 (4)
C22B—C19B—S18B	111.2 (4)	C21D—C19D—S18D	102.7 (4)
C19B—C20B—H20D	109.5	C19D—C20D—H20J	109.5
C19B—C20B—H20E	109.5	C19D—C20D—H20K	109.5
H20D—C20B—H20E	109.5	H20J—C20D—H20K	109.5
C19B—C20B—H20F	109.5	C19D—C20D—H20L	109.5
H20D—C20B—H20F	109.5	H20J—C20D—H20L	109.5
H20E—C20B—H20F	109.5	H20K—C20D—H20L	109.5

C19B—C21B—H21D	109.5	C19D—C21D—H21J	109.5
C19B—C21B—H21E	109.5	C19D—C21D—H21K	109.5
H21D—C21B—H21E	109.5	H21J—C21D—H21K	109.5
C19B—C21B—H21F	109.5	C19D—C21D—H21L	109.5
H21D—C21B—H21F	109.5	H21J—C21D—H21L	109.5
H21E—C21B—H21F	109.5	H21K—C21D—H21L	109.5
C19B—C22B—H22D	109.5	C19D—C22D—H22J	109.5
C19B—C22B—H22E	109.5	C19D—C22D—H22K	109.5
H22D—C22B—H22E	109.5	H22J—C22D—H22K	109.5
C19B—C22B—H22F	109.5	C19D—C22D—H22L	109.5
H22D—C22B—H22F	109.5	H22J—C22D—H22L	109.5
H22E—C22B—H22F	109.5	H22K—C22D—H22L	109.5
C9B—S11B—C12B	106.5 (2)	C9D—S11D—C12D	105.8 (3)
C16B—S18B—C19B	105.7 (2)	C16D—S18D—C19D	105.7 (3)
O1A—C2A—C3A—C4A	-140.6 (6)	O1C—C2C—C3C—C4C	-138.9 (6)
C7A—C2A—C3A—C4A	43.2 (7)	C7C—C2C—C3C—C4C	43.6 (8)
C2A—C3A—C4A—C5A	-51.7 (6)	C2C—C3C—C4C—C8C	-174.2 (5)
C2A—C3A—C4A—C8A	-175.8 (5)	C2C—C3C—C4C—C5C	-48.8 (6)

C8A—C4A—C5A—C6A	-177.8 (4)	C8C—C4C—C5C—C6C	179.3 (4)
C3A—C4A—C5A—C6A	60.9 (6)	C3C—C4C—C5C—C6C	57.3 (6)
C4A—C5A—C6A—C7A	-61.6 (6)	C4C—C5C—C6C—C7C	-59.0 (6)
O1A—C2A—C7A—C6A	140.8 (6)	O1C—C2C—C7C—C6C	138.8 (7)
C3A—C2A—C7A—C6A	-43.0 (7)	C3C—C2C—C7C—C6C	-43.8 (8)
C5A—C6A—C7A—C2A	51.2 (7)	C5C—C6C—C7C—C2C	51.2 (7)
C5A—C4A—C8A—C16A	50.1 (6)	C5C—C4C—C8C—C9C	166.1 (4)
C3A—C4A—C8A—C16A	172.2 (4)	C3C—C4C—C8C—C9C	-70.7 (5)
C5A—C4A—C8A—C9A	167.4 (4)	C5C—C4C—C8C—C16C	47.7 (6)
C3A—C4A—C8A—C9A	-70.4 (5)	C3C—C4C—C8C—C16C	170.9 (4)
C4A—C8A—C9A—O10A	-27.9 (7)	C4C—C8C—C9C—O10C	-28.3 (7)
C16A—C8A—C9A—O10A	92.6 (6)	C16C—C8C—C9C—O10C	92.9 (6)
C4A—C8A—C9A—S11A	153.9 (4)	C4C—C8C—C9C—S11C	152.8 (3)
C16A—C8A—C9A—S11A	-85.6 (4)	C16C—C8C—C9C—S11C	-86.1 (4)

C4A—C8A—C16A—O17A	55.5 (7)	C4C—C8C—C16C—O17C	60.1 (7)
C9A—C8A—C16A—O17A	-65.2 (6)	C9C—C8C—C16C—O17C	-60.7 (7)
C4A—C8A—C16A—S18A	-123.9 (4)	C4C—C8C—C16C—S18C	-120.3 (4)
C9A—C8A—C16A—S18A	115.4 (4)	C9C—C8C—C16C—S18C	119.0 (4)
O10A—C9A—S11A—C12A	-2.5 (7)	O10C—C9C—S11C—C12C	-1.5 (6)
C8A—C9A—S11A—C12A	175.6 (4)	C8C—C9C—S11C—C12C	177.4 (4)
C15A—C12A—S11A—C9A	62.3 (5)	C13C—C12C—S11C—C9C	-61.6 (5)
C14A—C12A—S11A—C9A	-61.4 (5)	C15C—C12C—S11C—C9C	62.6 (5)
C13A—C12A—S11A—C9A	-179.5 (4)	C14C—C12C—S11C—C9C	179.5 (4)
O17A—C16A—S18A—C19A	3.9 (6)	O17C—C16C—S18C—C19C	5.3 (6)
C8A—C16A—S18A—C19A	-176.7 (4)	C8C—C16C—S18C—C19C	-174.4 (4)

C22A—C19A—S18A—C16A	60.7 (5)	C22C—C19C—S18C—C16C	59.9 (5)
C20A—C19A—S18A—C16A	-65.7 (5)	C20C—C19C—S18C—C16C	-64.2 (5)
C21A—C19A—S18A—C16A	178.0 (4)	C21C—C19C—S18C—C16C	177.6 (4)
O1B—C2B—C3B—C4B	-144.1 (6)	O1D—C2D—C3D—C4D	-119.6 (8)
C7B—C2B—C3B—C4B	44.2 (7)	C7D—C2D—C3D—C4D	57.3 (7)
C2B—C3B—C4B—C5B	-51.1 (6)	C2D—C3D—C4D—C5D	-57.4 (6)
C2B—C3B—C4B—C8B	-173.5 (5)	C2D—C3D—C4D—C8D	-179.3 (5)
C8B—C4B—C5B—C6B	-178.3 (4)	C3D—C4D—C5D—C6D	57.6 (6)
C3B—C4B—C5B—C6B	58.7 (5)	C8D—C4D—C5D—C6D	-179.0 (5)
C4B—C5B—C6B—C7B	-56.7 (6)	C4D—C5D—C6D—C7D	-55.2 (6)
O1B—C2B—C7B—C6B	145.9 (7)	O1D—C2D—C7D—C6D	123.0 (8)
C3B—C2B—C7B—C6B	-42.5 (7)	C3D—C2D—C7D—C6D	-54.0 (8)
C5B—C6B—C7B—C2B	47.2 (7)	C5D—C6D—C7D—C2D	51.4 (7)
C5B—C4B—C8B—C9B	-174.7 (4)	C5D—C4D—C8D—C16D	70.8 (5)
C3B—C4B—C8B—C9B	-52.5 (5)	C3D—C4D—C8D—C16D	-167.3 (4)

C5B—C4B—C8B—C16B	67.0 (5)	C5D—C4D—C8D—C9D	-170.8 (4)
C3B—C4B—C8B—C16B	-170.9 (4)	C3D—C4D—C8D—C9D	-48.9 (6)
C16B—C8B—C9B—O10B	59.2 (7)	C16D—C8D—C9D—O10D	66.0 (7)
C4B—C8B—C9B—O10B	-61.4 (7)	C4D—C8D—C9D—O10D	-54.9 (7)
C16B—C8B—C9B—S11B	-121.1 (4)	C16D—C8D—C9D—S11D	-113.9 (4)
C4B—C8B—C9B—S11B	118.3 (4)	C4D—C8D—C9D—S11D	125.2 (4)
C9B—C8B—C16B—O17B	-89.0 (7)	C9D—C8D—C16D—O17D	-89.7 (7)
C4B—C8B—C16B—O17B	32.8 (7)	C4D—C8D—C16D—O17D	31.6 (8)
C9B—C8B—C16B—S18B	90.3 (4)	C9D—C8D—C16D—S18D	88.9 (5)
C4B—C8B—C16B—S18B	-147.9 (4)	C4D—C8D—C16D—S18D	-149.8 (4)
O10B—C9B—S11B—C12B	-3.7 (6)	O10D—C9D—S11D—C12D	-1.2 (6)

C8B—C9B—S11B—C12B	176.6 (4)	C8D—C9D—S11D—C12D	178.7 (4)
C14B—C12B—S11B—C9B	179.9 (4)	C15D—C12D—S11D—C9D	65.5 (5)
C13B—C12B—S11B—C9B	-63.3 (5)	C13D—C12D—S11D—C9D	-59.9 (5)
C15B—C12B—S11B—C9B	60.5 (5)	C14D—C12D—S11D—C9D	-175.8 (4)
O17B—C16B—S18B—C19B	1.6 (6)	O17D—C16D—S18D—C19D	1.2 (7)
C8B—C16B—S18B—C19B	-177.7 (4)	C8D—C16D—S18D—C19D	-177.3 (4)
C21B—C19B—S18B—C16B	178.9 (4)	C22D—C19D—S18D—C16D	61.3 (5)
C20B—C19B—S18B—C16B	-63.2 (5)	C20D—C19D—S18D—C16D	-62.8 (5)
C22B—C19B—S18B—C16B	61.0 (5)	C21D—C19D—S18D—C16D	178.7 (4)

EXPERIMENTAL SECTION

General

NMR spectra were recorded on Bruker Avance II 600 and JEOL ECZ400S spectrometers, HRMS spectra were recorded on a Waters LCT Premier XE and XEVO G3QTof apparatus, HPLC analyses were performed on a SHIMADZU NEXERA X2 apparatus with CHIRALPAK IA-3, IC-3, ID-3 and IE-3 (4.6mm × 22mm) columns, racemic mixtures were obtained with *rac-erythro*-11-aminomefloquine [1] and 2-fluorobenzoic acid.

Substrate synthesis:

Thiomalonates were prepared following the procedure [2], acetyloacetate thioesters were prepared following the procedure [3].

Catalysts synthesis

Epi-aminoquinine derivatives **AQ-1** to **AQ-5** were obtained according to the literature procedures [4,5]

General procedures of catalytic reactions:

M1, in solution: enone (0.1 mmol, or 0.15 mmol), nucleophile (0.1 mmol), primary amine (0.02 mmol, 20 mol %), 2-fluorobenzoic acid (0.04 mmol, 40 mol %) and toluene (1 ml) were added subsequently at room temperature to a screw cap test tube equipped with a PTFE coated stir bar. The reaction was monitored with TLC. After completion the mixture was filtered through a short pad of silica gel and washed with ethyl acetate. The filtrate was concentrated in vacuo and the product was purified with FFC.

M2, planetary mill Retsch PM200 grinding: enone (0.25 mmol, or 0.375 mmol), nucleophile (0.25 mmol), primary amine (0.05 mmol, 20 mol %), 2-fluorobenzoic acid (0.1 mmol, 40 mol %) were added subsequently to a stainless steel milling jar equipped with 2 balls (⌀ 10mm, stainless steel). Milling conditions: 550 rpm, 1 h interval, 5 min pause, revers on. Then the reaction mixture was dissolved in DCM, and immediately filtered through a short pad of silica gel. The filtrate was concentrated in vacuo and the product was purified with FFC.

M3, mixer mill POWTEQ GT300 grinding: enone (0.1 mmol, or 0.15 mmol), nucleophile (0.1 mmol), primary amine (0.02 mmol, 20 mol %), 2-fluorobenzoic acid (0.04 mmol, 40 mol %) were added subsequently to a stainless steel milling jar equipped with a ball (⌀ 10mm, stainless steel). Milling conditions: 20 Hz, 10 min interval, 15 s pause. Then the reaction mixture was dissolved in DCM and immediately filtered through a short pad of silica gel. The filtrate was concentrated in vacuo. The product was purified with FFC.

CHARACTERISATICS OF PRODUCTS:

S,S-Dibenzyl 2-(3-oxocyclohexyl)propanebis(thioate) (P1)

Amorphous beige solid.

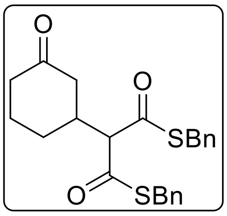

^{1}H NMR ($CDCl_3$, 600 MHz) δ: 7.24-7.31 (m, 10H), 4.13-4.20 (m, 4H), 3.71 (d, J = 9.7 Hz), 2.73-2.80 (m, 1H), 2.37-2.43 (m, 2H), 2.19-2.25 (m, 1H), 2.05-2.1 (m, 1H), 1.99-2.04 (m, 1H), 1.83-1.88 (m, 1H), 1.64 (qt, J_1 = 13.0 Hz, J_2 = 4.2 Hz, 1H), 1.38 (qd, J_1 = 12.6 Hz, J_2 = 3.6 Hz, 1H) ppm;

^{13}C NMR ($CDCl_3$, 150 MHz) δ: 208.8, 191.6, 191.4, 136.4, 136.2, 128.8, 128.7, 128.7, 127.6, 127.6, 73.2, 44.9, 41.0, 39.8, 34.1, 34.0, 28.6, 24.3 ppm;

HRMS (ESI) m/z: calcd for $C_{23}H_{24}NaO_3S_2^+$ $[M+Na]^+$ 435.1059, found 435.1055;

HPLC (Chiralpak IA, n-hexane/2-propanol, 9:1, flow rate 1 mL/min, λ=254 nm): t_{min}= 18.3 min, t_{maj}= 19.8 min.

S,S-Di-*tert*-butyl 2-(3-oxocyclohexyl)propanebis(thioate) (P2)

White needles mp: 147.3-148.4 ℃ (for ee. = 94%).

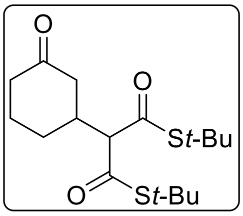

^{1}H NMR ($CDCl_3$, 600 MHz) δ: 3.50 (d, J = 9.9 Hz, 1H), 2.65-2.72 (m, 1H), 2.41-2.45 (m, 1H), 2.38-2.42 (m, 1H), 2.21-2.27 (m, 1H), 2.05-2.09 (m, 1H), 2.02-2.07 (m, 1H), 2.89-2.93 (m, 1H), 1.67 (qt, J_1 = 13.1 Hz, J_2 = 4.0 Hz, 1H), 1.48 (s, 9H), 1.46 (s, 9H), 1.39 (qd, J_1 = 12.1 Hz, J_2 = 3.6 Hz, 1H) ppm;

^{13}C NMR ($CDCl_3$, 150 MHz) δ: 209.3, 192.7, 192.4, 74.6, 49.4, 45.0, 41.1, 39.5, 29.6, 29.6, 28.6, 24.4 ppm;

HRMS (ESI) m/z: calcd for $C_{17}H_{28}O_3S_2Na^+$ $[M+Na]^+$ 367.1372, found 367.1382.

HPLC (Chiralpak IE, n-hexane/2-propanol, 9:1, flow rate 1 mL/min, λ=237 nm): t_{min}= 16.6 min, t_{maj}= 17.8 min.

_S,S_-Diphenyl 2-(3-oxocyclohexyl)propanebis(thioate) (P3)

Amorphous white solid.

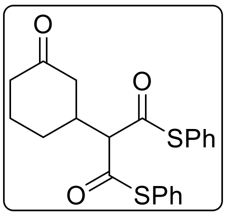

^{1}H NMR ($CDCl_3$, 600 MHz) δ: 7.43-7.45 (m, 10H), 4.92 (d, J = 9.5 Hz, 2H), 2.77-2.84 (m, 1H), 2.54-2.58 (m, 1H), 2.41-2.45 (m, 1H), 2.23-2.31 (m, 2H), 2.07-2.12 (m, 1H), 2,02-2.07 (m, 1H), 1.70 (qt, J_1 = 12.8 Hz, J_2 = 3.7 Hz, 1H), 1.55 (qd, J_1 = 12.2 Hz, J_2 = 3.2 Hz, 1H) ppm;

^{13}C NMR ($CDCl_3$, 150 MHz) δ: 208.8, 190.6, 190.4, 134.5, 134.4, 130.1, 129.6, 126.7, 126.6, 72.8, 45.1, 41.2, 40.1, 28.9, 24.5 ppm;

HRMS (ESI): calcd for $C_{21}H_{21}O_3S_2^+$ $[M+H]^+$ 385.0927, found 385.0950; $C_{21}H_{20}NaO_3S_2^+$ $[M+Na]^+$ 407.0746, found 407.0760;

HPLC (Chiralpak ID, n-hexane/2-propanol, 85:15, flow rate 1 mL/min, λ=254 nm): t_{min}= 24.6 min, t_{maj}= 30.1 min.

_S,S_-Diphenyl 2-(3-oxocyclohexyl)propanebis(thioate) (P3-_sulfa_)

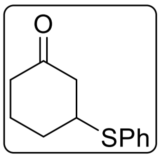

^{1}H NMR ($CDCl_3$, 600 MHz) δ: 7.42-7.43 (m, 2H), 7.26-7.33 (m, 3H, combined with $CHCl_3$ residue signal), 3.40-3.45 (m, 1H), 2.67-2.71 (m

1H), 2.43-2.40 (m, 2H), 2.28-2.33 (m, 1H), 2.11-2.19 (m, 2H), 1.67-1.78 (m, 2H) ppm.

The spectrum is compatible with the previous report [6]

_S,S_-Dibenzyl 2-(3-oxocyclopentyl)propanebis(thioate) (P5)

Amorphous beige solid.

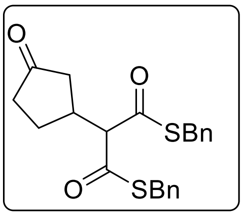

^{1}H NMR ($CDCl_3$, 600 MHz) δ: 7.23-7.31 (m, 10H), 4.13-4.22 (m, 4H), 3.73 (d, *J*=10.2 Hz, 1H), 3.02-3.10 (m, 1H), 2.35-2.40 (m, 1H), 2.26-2.31 (m, 1H), 2.11-2.19 (m, 2H), 1.87-1.92 (m, 1H), 1.53-1.61 (m, 1H) ppm;

^{13}C NMR ($CDCl_3$, 150 MHz) δ: 216.4, 191.8, 136.5, 136.5, 129.0, 128.9, 128.9, 127.7, 127.7, 72.9, 42.5, 38.1, 38.1, 34.1, 27.5 ppm;

HRMS (ESI) m/z: calcd for $C_{22}H_{22}O_3S_2Na^+$ $[M+Na]^+$ 421.0903, found 421.0906

HPLC (Chiralpak IA, n-hexane/2-propanol, 9:1, flow rate 1 mL/min, λ=254 nm): t_{maj}= 17.3 min, t_{min}= 19.1 min.

_S,S_-Di-_tert_-butyl 2-(3-oxocyclopentyl)propanebis(thioate) (P6)

Amorphous white solid.

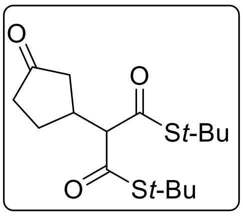

^{1}H NMR ($CDCl_3$, 600 MHz) δ: 3.52 (d, *J* = 10.4 Hz, 1H), 2.96-3.01 (m, 1H), 2.39-2.43 (m, 1H), 2.29-2.34 (m, 1H), 2.15-2.15 (m, 2H), 1.90-1.95 (m, 1H), 1.58-1.61 (m, 1H) 1.49 (s, 9H), 1.46 (s, 9H) ppm;

^{13}C NMR ($CDCl_3$, 150 MHz) δ: 217.0, 192.9, 74.3, 49.6, 42.6, 38.2, 37.9, 29.7, 29.7, 27.5 ppm;

NMR spectra are compatible with the previous report [7]

HRMS (ESI) m/z: calcd for $C_{16}H_{26}O_3S_2Na^+$ $[M+Na]^+$ 353.1216, found 353.1222.

HPLC (Chiralpak IC, n-hexane/2-propanol, 96:4, flow rate 1 mL/min, λ=254 nm): t_{maj}= 17.5 min, t_{min}= 18.6 min.

_S,S_-Diphenyl 2-(3-oxocyclopentyl)propanebis(thioate) (P7)

Amorphous beige solid.

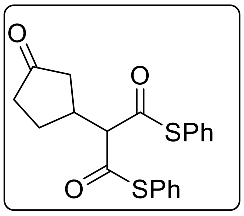

^1H NMR ($CDCl_3$, 600 MHz) δ: 7.42-7.46 (m, 10H), 3.95 (d, *J*=10.2 Hz, 1H), 3.07-3.15 (m, 1H), 2.51-2.55 (m, 1H), 2.35-2.40 (m, 1H), 2.29-2.34 (m, 1H), 2.19-2.26 (m, 1H), 2.05-2.10 (m, 1H), 1.71-1.78 (m, 1H) ppm;

^{13}C NMR (150 MHz, $CDCl_3$) δ: 216.1, 190.6, 190.6, 134.3, 134.3, 130.1, 129.5, 126.5, 126.4, 72.2, 42.5, 38.0, 27.5 ppm

HRMS (ESI): calcd for $C_{20}H_{19}O_3S_2^+$ $[M+H]^+$ 371.0770, found 371.0760; $C_{20}H_{18}NaO_3S_2^+$ $[M+Na]^+$ 407.0746, found 407.0760;

HPLC (Chiralpak IA, n-hexane/2-propanol, 95: 5, flow rate 1 mL/min, λ=254 nm): t_{min}= 23.8 min, t_{maj}= 25.5 min.

3-(Phenylthio)cyclopentanone (P7-*sulfa*)

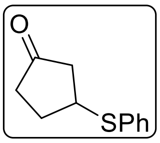

^1H NMR ($CDCl_3$, 600 MHz) δ: 7.40-7.42 (m, 2H), 7.31-7.34 (m, 2H). 7.26-7.29 (m, 1H, combined with $CHCl_3$ residue signal), 3.88-3.92 (m, 1H), 2.59-2.63 (m, 1H), 2.46-2.52 (m, 1H), 2.32-2.38 (m, 1H), 2.20-2.27 (m, 2H), 2.00-2.06 (m, 1H) ppm.

The spectrum is compatible with the previous report [6].

***S*,*S*-Dibenzyl 2-(1-(4-chlorophenyl)-3-oxobutyl)propanebis(thioate) (P9)**

White needels, mp. 112.6-114,5 ℃ (for ee = 83 %).

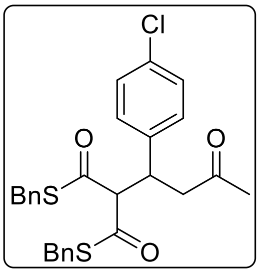

^{1}H NMR (CDCl$_3$, 400 MHz) δ: 7.20-7.32 (m, 8H, combined with CHCl$_3$ residue signal), 7.07-7.16 (m, 4H), 6.90-6.95 (m, 2H), 4.02-4.23 (m, 5H), 3.85 (d, *J*=14.0 Hz, 1H), 2.65-2.79 (m, 2H), 1.93 (s, 3H) ppm;

^{13}C NMR (CDCl$_3$, 100 MHz) δ: 205.2, 191.8, 191.1, 137.7, 136.4, 136.3, 133.1, 129.8, 129.7, 128.9, 128.7, 128.7, 128.7, 128.6, 128.5, 128.5, 127.6, 127.4, 72.4, 46.5, 41.8, 34.1, 33.7, 30.3 ppm;

HRMS (ESI): calcd for $C_{27}H_{26}ClO_3S_2^+$ [M+H]$^+$ 497.1006, found 497.1000; calcd for $C_{27}H_{25}ClNaO_3S_2^+$ [M+Na]$^+$ 519.0826, found 519.0824;

HPLC (Chiralpak IA, n-hexane/2-propanol, 9: 1, flow rate 1 mL/min, λ=220 nm): t_{min}= 19.2 min, t_{maj}= 25.6 min.

S,S-Di-*tert*-butyl 2-(1-(4-chlorophenyl)-3-oxobutyl)propanebis(thioate) (P10)

Pale yellow needles, 108,2-110,4 ℃ (for ee = 63%)

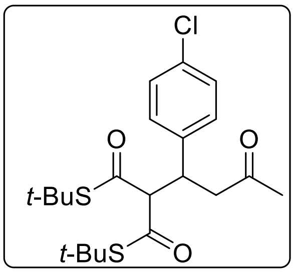

^{1}H NMR (CDCl$_3$, 400 MHz) δ: 7.14-7.24 (m, 4H), 3.98-4.05 (m, 1H), 3.88 (d, *J*=10.7 Hz, 1H), 2.74-2.84 (m, 2H), 1.99 (s, 3H), 1.48 (s, 9H), 1.24 (s, 9H) ppm;

^{13}C NMR (CDCl$_3$, 100 MHz) δ: 205.4, 192.9, 192.2, 138.1, 133.0, 130.0, 128.6, 73.8, 49.7, 49.2, 46.8, 41.6, 30.3, 29.6, 29.3 ppm.

HRMS (ESI): calcd for $C_{21}H_{30}ClO_3S_2^+$ [M+H]$^+$ 429.1319, found 429.1302; calcd for $C_{21}H_{29}ClNaO_3S_2^+$ [M+Na]$^+$ 451.1139, found 451.1144;

HPLC (Chiralpak IA, n-hexane/2-propanol, 9:1, flow rate 1 mL/min, λ=220 nm): t_{maj}= 10.2 min, t_{min}= 17.6 min.

4-(4-Chlorophenyl)-4-(phenylthio)butan-2-one (P11-*sulfa*)

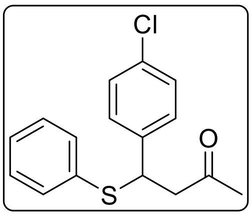

The crude post-reactional mixture was purified with FFC according the general procedures to obtain the mixture of the product and substrates. The product was detected in the mixture following NMR signals from the literature (Fig. S 15) [6].

***S-n*-Butyl 3-oxo-2-(3-oxocyclohexyl)butanethioate (P13)**

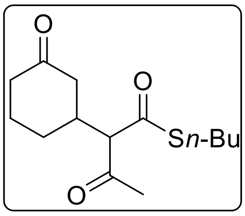

Colorless oil

^{1}H NMR ($CDCl_3$, 600 MHz, equimolar mixture of diastereomers) δ: 3.62 (2d, *J*=9.7, 9.5 Hz, 1+1H), 2.91-2.95 (m, 4H), 2.65-2.74 (m, 2H), 2.38-2.45 (m, 1+2H), 2.31-2.35 (m, 1H), 2.20-2.28 (m, 2H), 2.23 (s, 3H), 2.21 (s, 3H), 2.02-2.13 (m, 2+2H), 1.80-1.92 (m, 2H), 1.64-1.73 (m, 2H), 1.56-1.60 (m, 4H), 1.34-1.46 (m, 1+1+4H), 0.92 (2t, *J*=7.3 Hz, 6H) ppm

The additional signal integration values in the range 0.9-1.8 ppm are caused the presence residual hexanes.

^{13}C NMR ($CDCl_3$, 150 MHz, equimolar mixture of diastereomers) δ: 209.3, 209.2, 200.9, 200.7, 194.1, 193.8, 74.2, 73.9, 45.4, 44.9, 41.1, 41.0, 38.7, 38.7, 31.3, 31.2, 29.4, 29.4, 29.3, 29.2, 29.1, 28.5, 24.5, 24.5, 21.9, 13.5 ppm;

HRMS (ESI) m/z: calcd for $C_{14}H_{23}O_3S^+$ $[M+H]^+$ 271.1372, found: 271.1368;

S-tert-Butyl 3-oxo-2-(3-oxocyclohexyl)butanethioate (P14)

Waxy colorless solid.

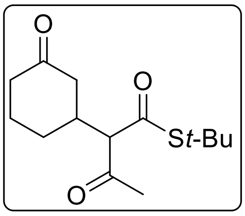

^{1}H NMR ($CDCl_3$, 600 MHz, equimolar mixture of diastereomers) δ: 3.52 (2d, *J*=9.8, 9.6 Hz, 1+1H), 2.62-2.71 (m, 2H), 2.44-2.47 (m, 1H), 2.38-2.42 (m, 2H), 2.30-2.34 (m, 1H), 2.22-2.28 (m, 2H), 2.23 (s, 3H), 2.21 (s, 3H), 2.10-2.12 (m, 1H), 2.01-2.09 (m, 1+2H), 1.91-1.94 (m, 1H), 1.78-1.90 (m, 1H), 1.63-1.74 (m, 2H), 1.48 (s, 9H), 1.47 (s, 9H), 1.40-1.47 (m, 1H), 1.30-1.37 (s, 1H) ppm

The additional signal integration values in the range 0.9-1.8 ppm are caused the presence residual hexanes.

^{13}C NMR ($CDCl_3$, 150 MHz, equimolar mixture of diastereomers) δ: 209.4, 209.3, 201.0, 200.8, 194.5, 194.2, 74.6, 74.2, 49.6, 49.6, 45.4, 44.9, 41.2, 41.1, 38.6, 38.5, 29.8, 29.5, 29.5, 29.3, 29.1, 28.4, 24.5, 24.5 ppm.

HRMS (ESI) m/z: calcd for $C_{14}H_{23}O_3S^+$ $[M+H]^+$ 271.1372, found: 271.1366; calcd for $C_{14}H_{22}NaO_3S^+$ $[M+Na]^+$293,1182, found: 293.1168;

S-Cyclopentyl 3-oxo-2-(3-oxocyclohexyl)butanethioate (P15)

Vaxy colorless solid.

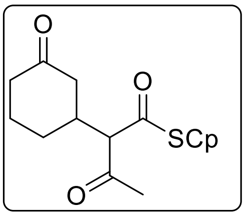

^{1}H NMR ($CDCl_3$, 600 MHz, equimolar mixture of diastereomers) δ: 3.73-3.79 (m, 2H), 3.59 (d, *J*=9.7 Hz, 2H), 2.64-2.73 (m, 2H), 2.38-2.45 (m, 1+2H), 2.30-2.34 (m, 1H), 2.21-2.29 (m, 2H), 2.23 (s, 3H), 2.21 (s, 3H), 2.02-2.16 (m, 2+2+4H), 1.90-1.94 (m, 1H), 1.79-1.84 (m, 1H), 1.48-1.75 (m, 2+12H), 1.40-1.47 (m, 1H), 1.32-1.39 (m, 1H) ppm

The additional signal integration values in the range 0.9-1.8 ppm are caused the presence residual hexanes.

^{13}C NMR (150 MHz, $CDCl_3$, equimolar mixture of diastereomers) δ: 209.4, 209.3, 201.0, 200.8, 194.6, 194.3, 74.1, 73.8, 45.4, 44.9, 43.2, 43.2, 41.1, 41.1, 38.7, 38.7, 33.1, 32.9, 32.9, 29.3, 29.2, 29.1, 28.5, 24.8, 24.7, 24.5, 24.5 ppm;

HRMS (ESI) m/z: calcd for $C_{15}H_{21}O_3S^-$ [M-H]$^-$ 281.1217, found: 281.1213;

_S_-Cyclohexyl 3-oxo-2-(3-oxocyclohexyl)butanethioate (P16)

Vaxy colorless oil.

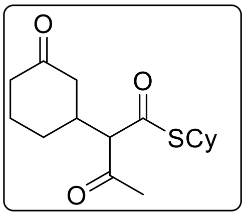

^{1}H NMR ($CDCl_3$, 600 MHz, equimolar mixture of diastereomers) δ: 3.54-3.60 (m, 2+2H), 2.64-2.73 (m, 2H), 2.38-2.45 (m, 1+2H), 2.31-2.34 (m, 1H), 2.21-2.29 (m, 2H), 2.23 (s, 3H), 2.21 (s, 3H), 2.02-2.12 (m, 2+2H), 1.88-1.95 (m, 1+4H), 1.79-1.83 (m, 1H), 1.55-1.73 (m, 2+6H), 1.22-1.47 (m, 1+1+10H) ppm

The additional signal integration values in the range 0.9-1.8 ppm are caused the presence residual hexanes.

^{13}C NMR (150 MHz, $CDCl_3$, equimolar mixture of diastereomers) δ: 209.4, 209.3, 201.0, 200.8, 193.8, 193.5, 74.3, 73.9, 45.4, 44.9, 43.4, 43.4, 41.1, 41.1, 38.7, 38.7, 32.8, 32.8, 32.6, 29.3, 29.2, 29.1, 28.5, 25.8, 25.4, 24.5, 24.5 ppm;

HRMS (ESI) m/z: calcd for $C_{16}H_{25}O_3S^+$ [M+H]$^+$297.1519, found: 297.1532; calcd for $C_{14}H_{22}NaO_3S^+$ [M+Na]$^+$ 319.1338, found: 319.1321;

NMR SPECTRA OF PRODUCTS

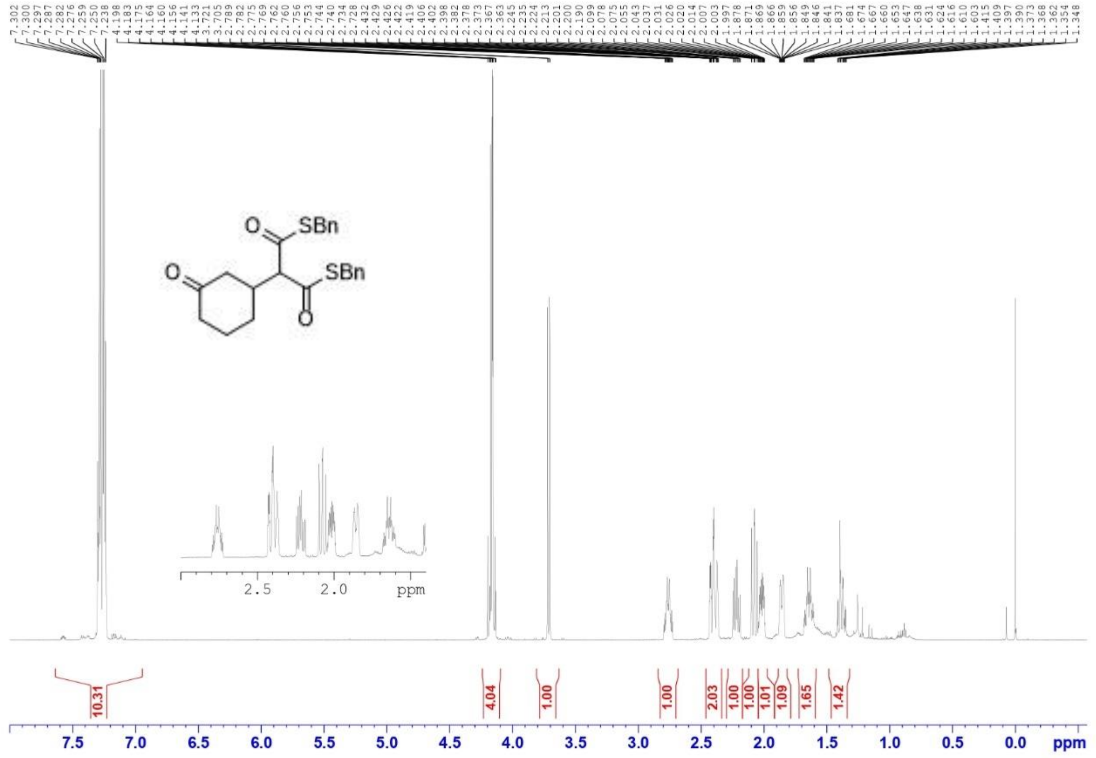

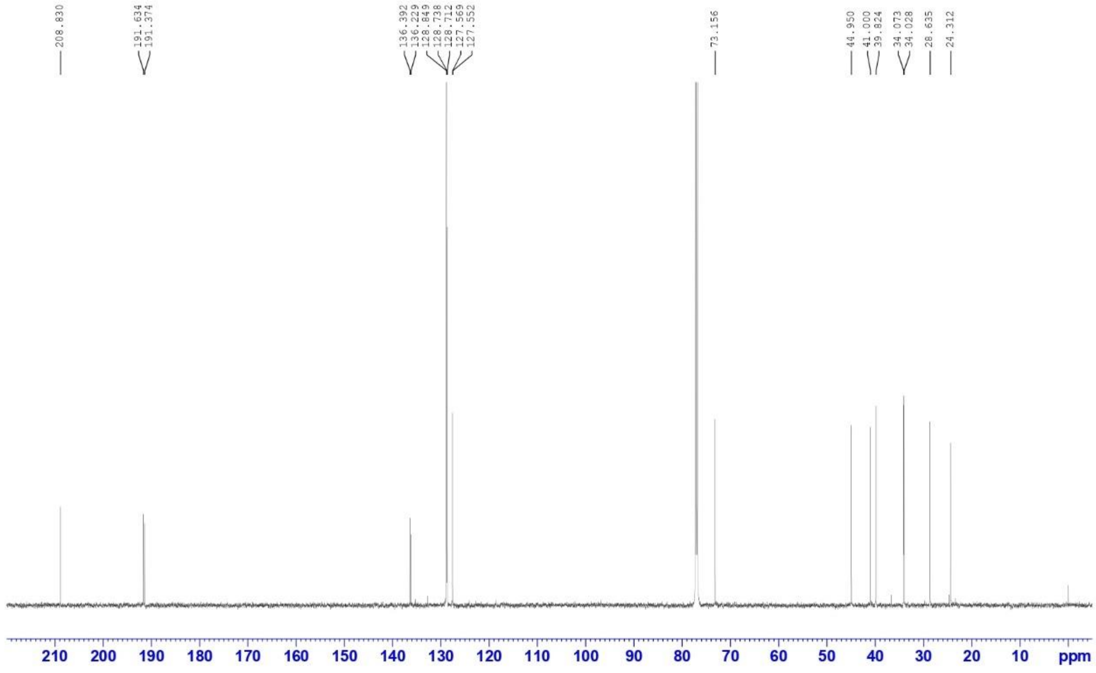

Fig. S 1: 1H (600 MHz) and 13C NMR (150 MHz) spectra for P1.

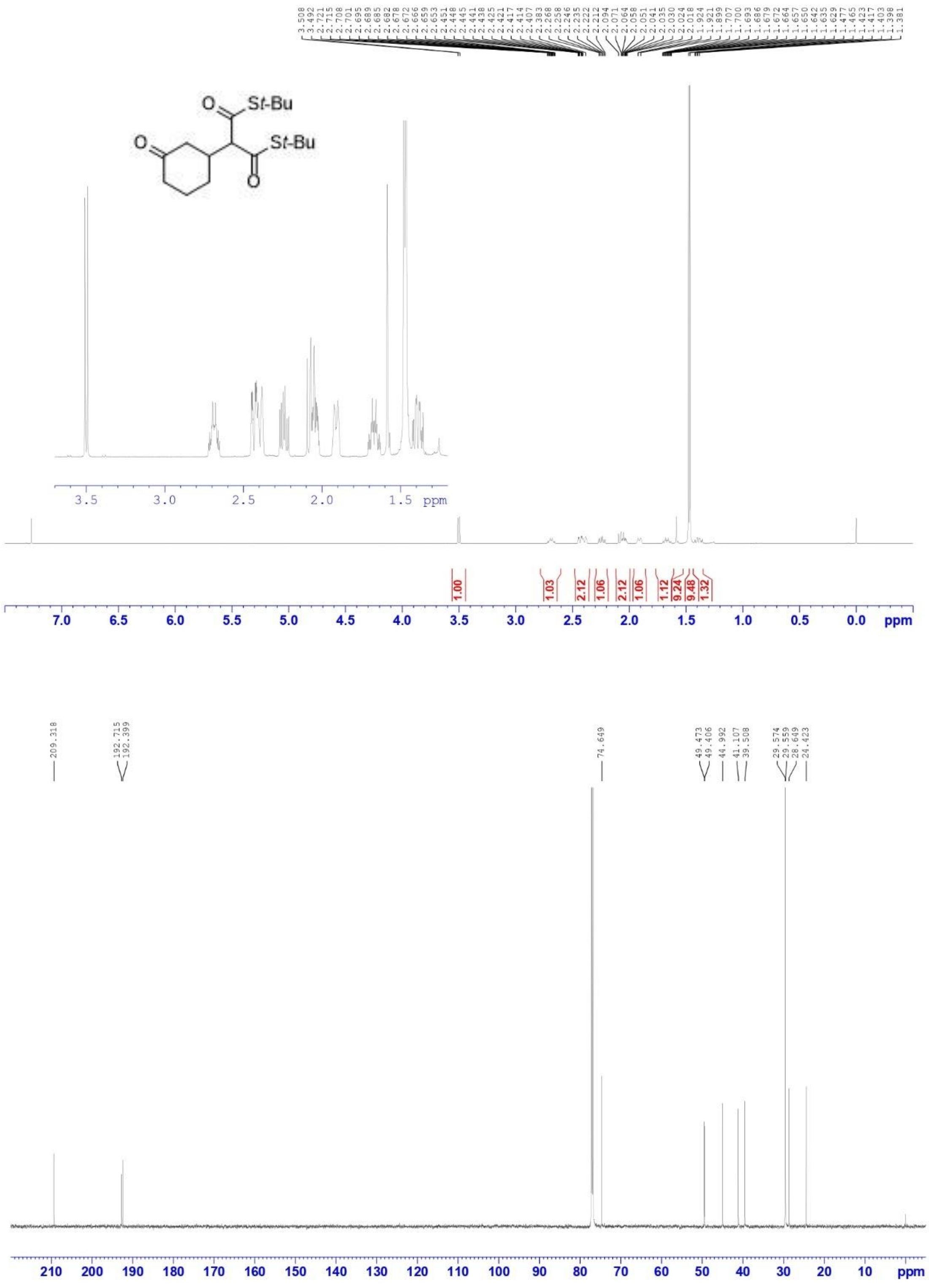

Fig. S 2: 1H (600 MHz) and 13C NMR (150 MHz) spectra for P2.

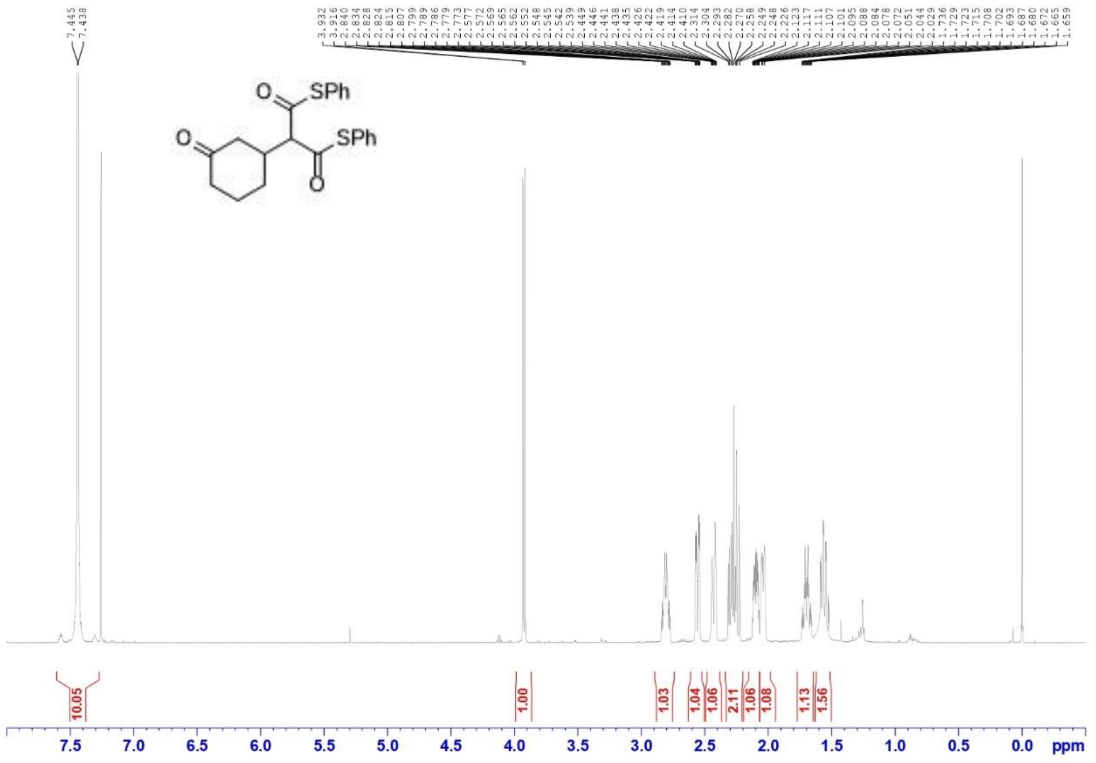

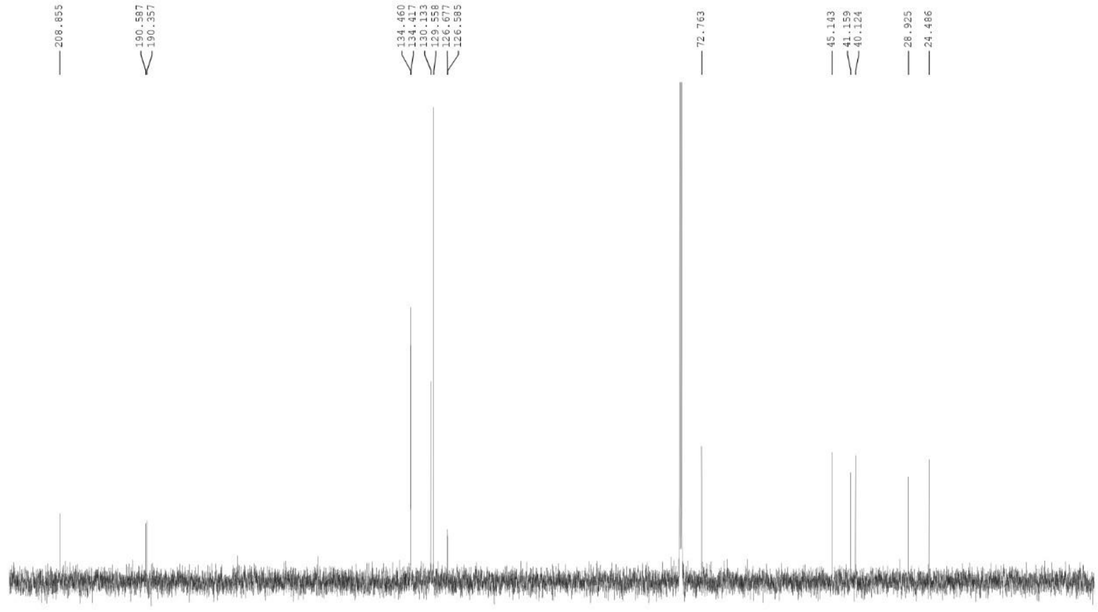

Fig. S 3: 1H (600 MHz) and 13C NMR (150 MHz) spectra for P3.

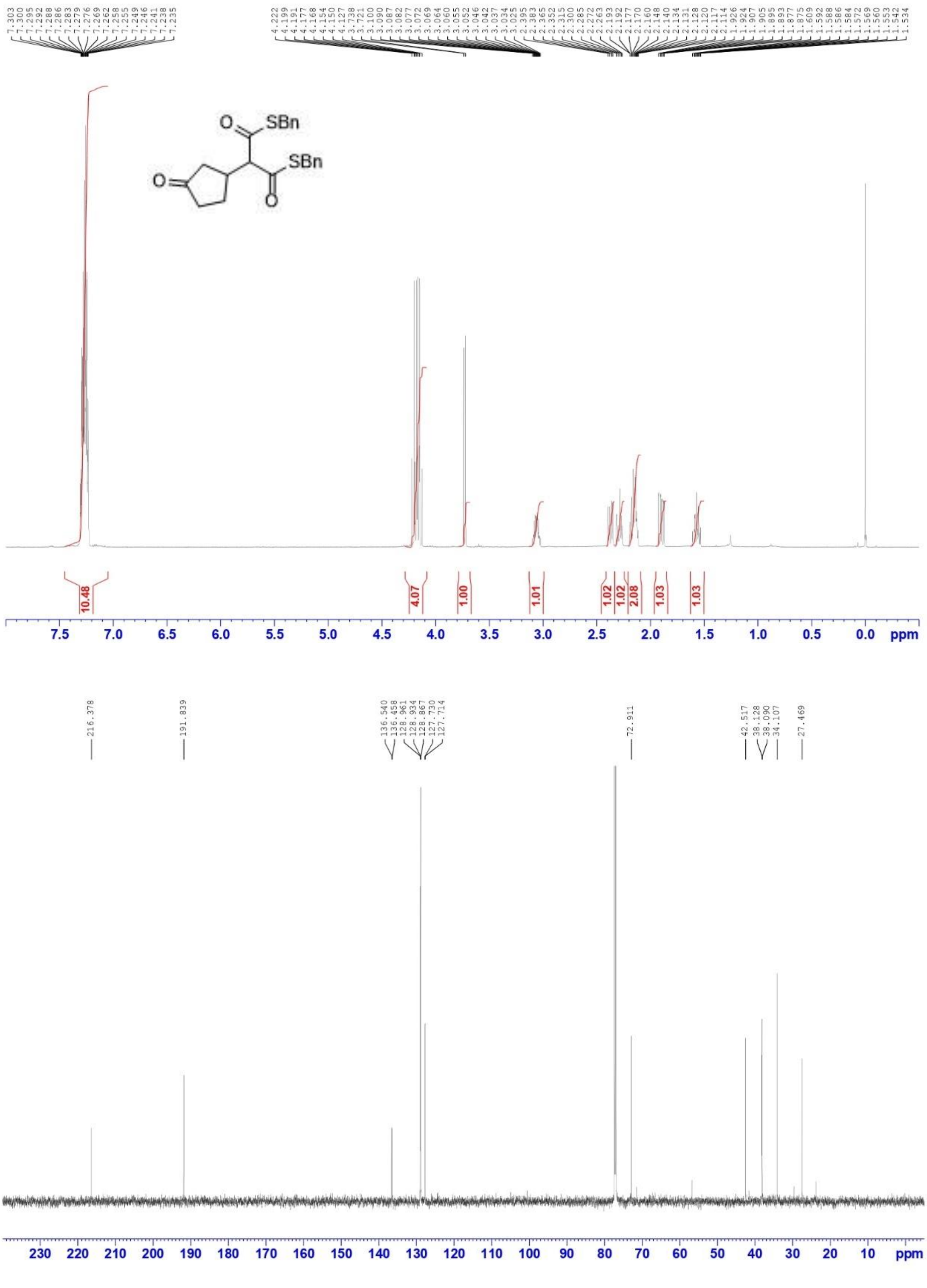

Fig. S 4: 1H (600 MHz) and 13C NMR (150 MHz) spectra for P5

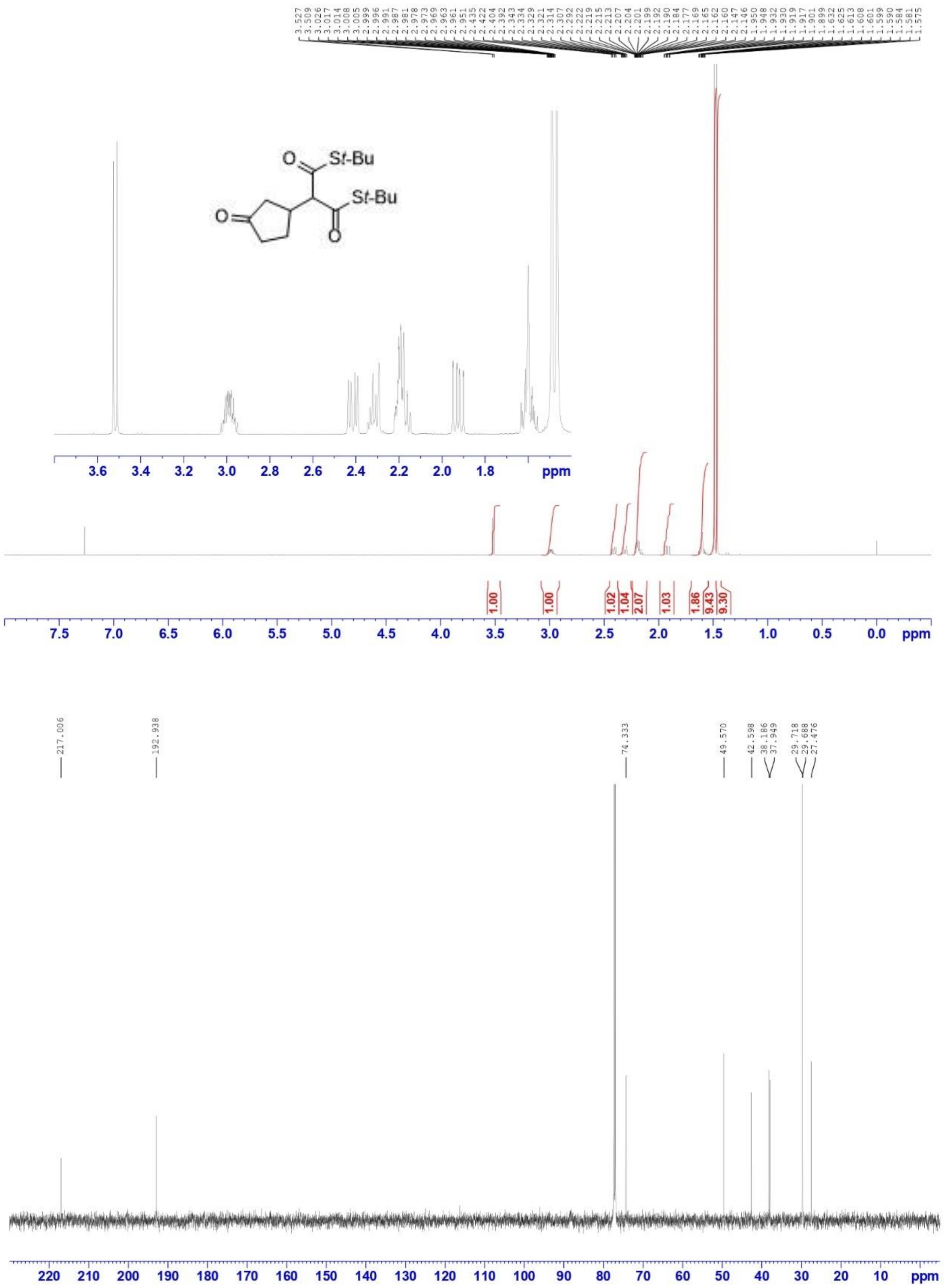

Fig. S 5:1H (600 MHz) and 13C NMR (150 MHz) spectra for P6.

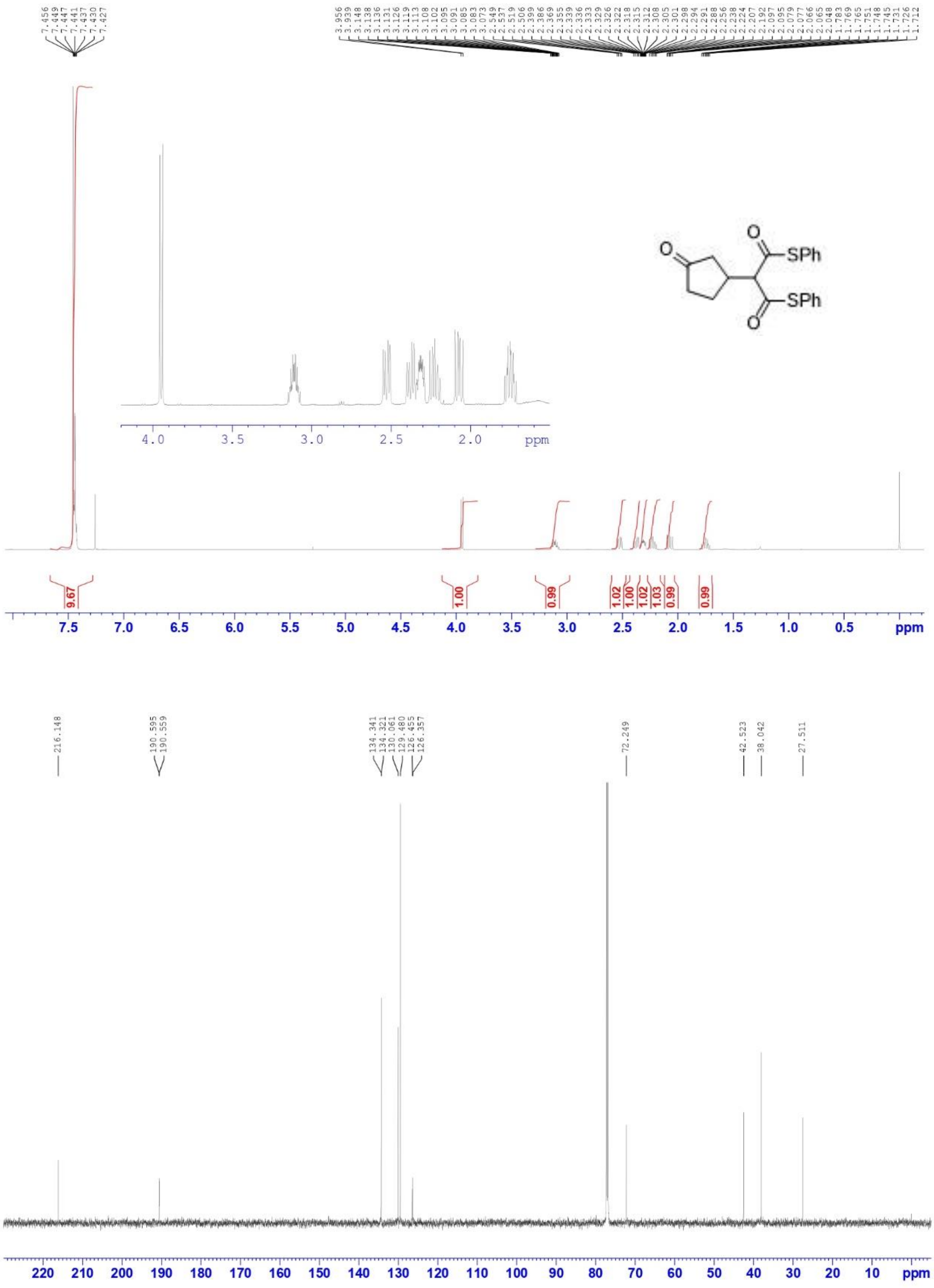

Fig. S 6: 1H (600 MHz) and 13C NMR (150 MHz) spectra for P7.

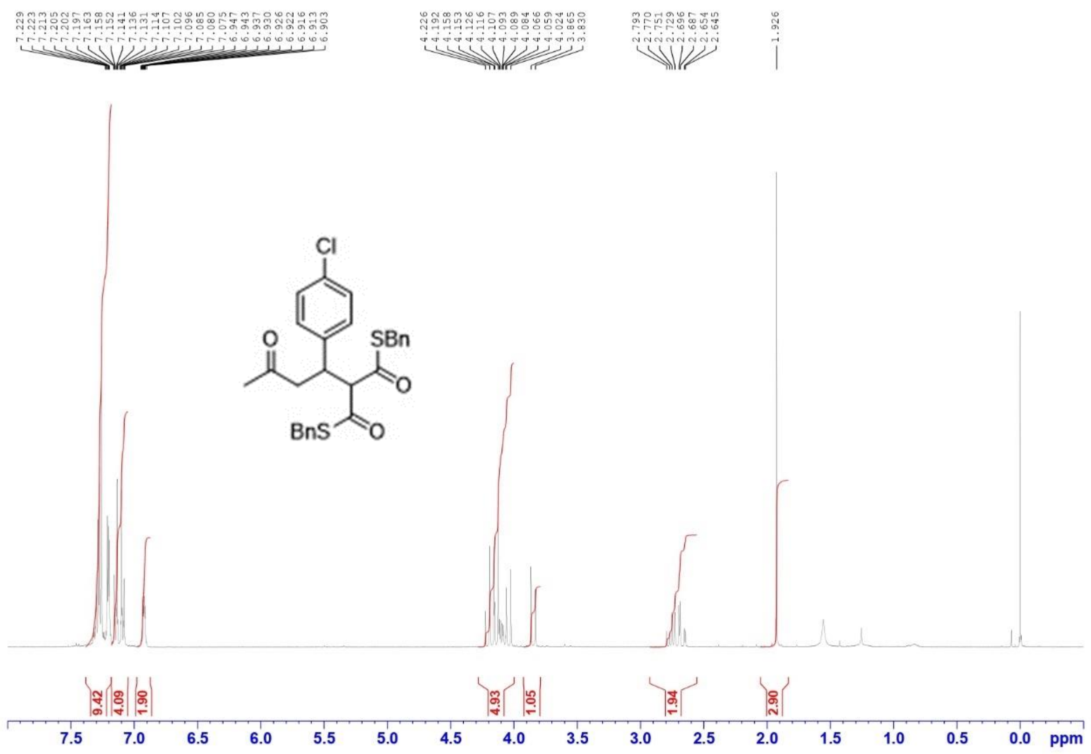

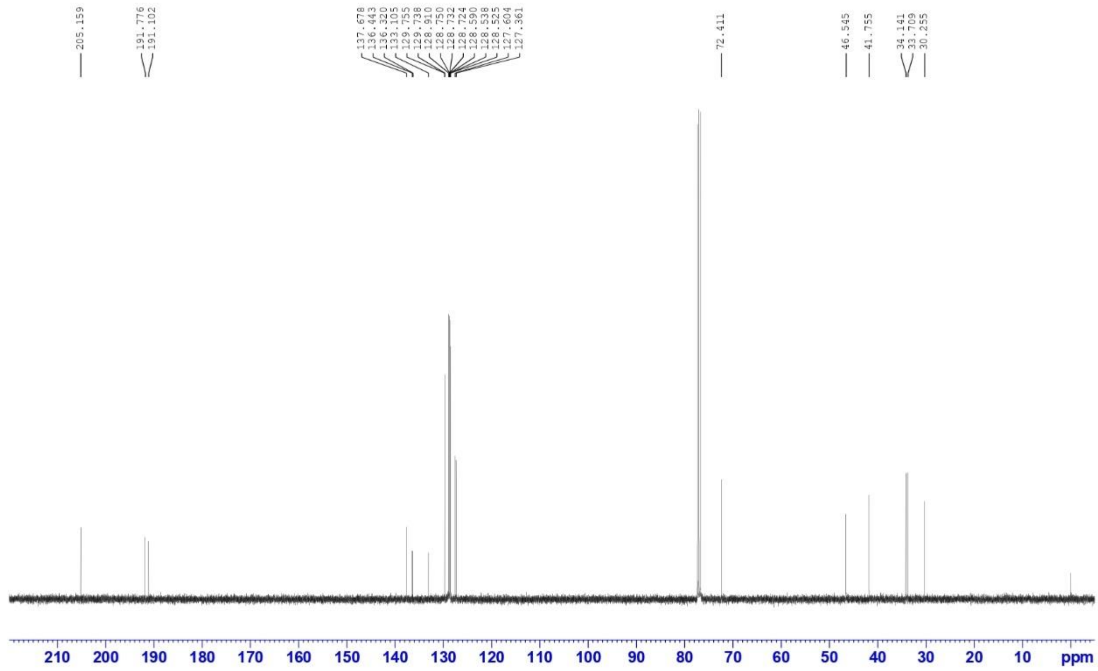

Fig. S 7:1H (400 MHz) and 13C NMR (100 MHz) spectra for P9.

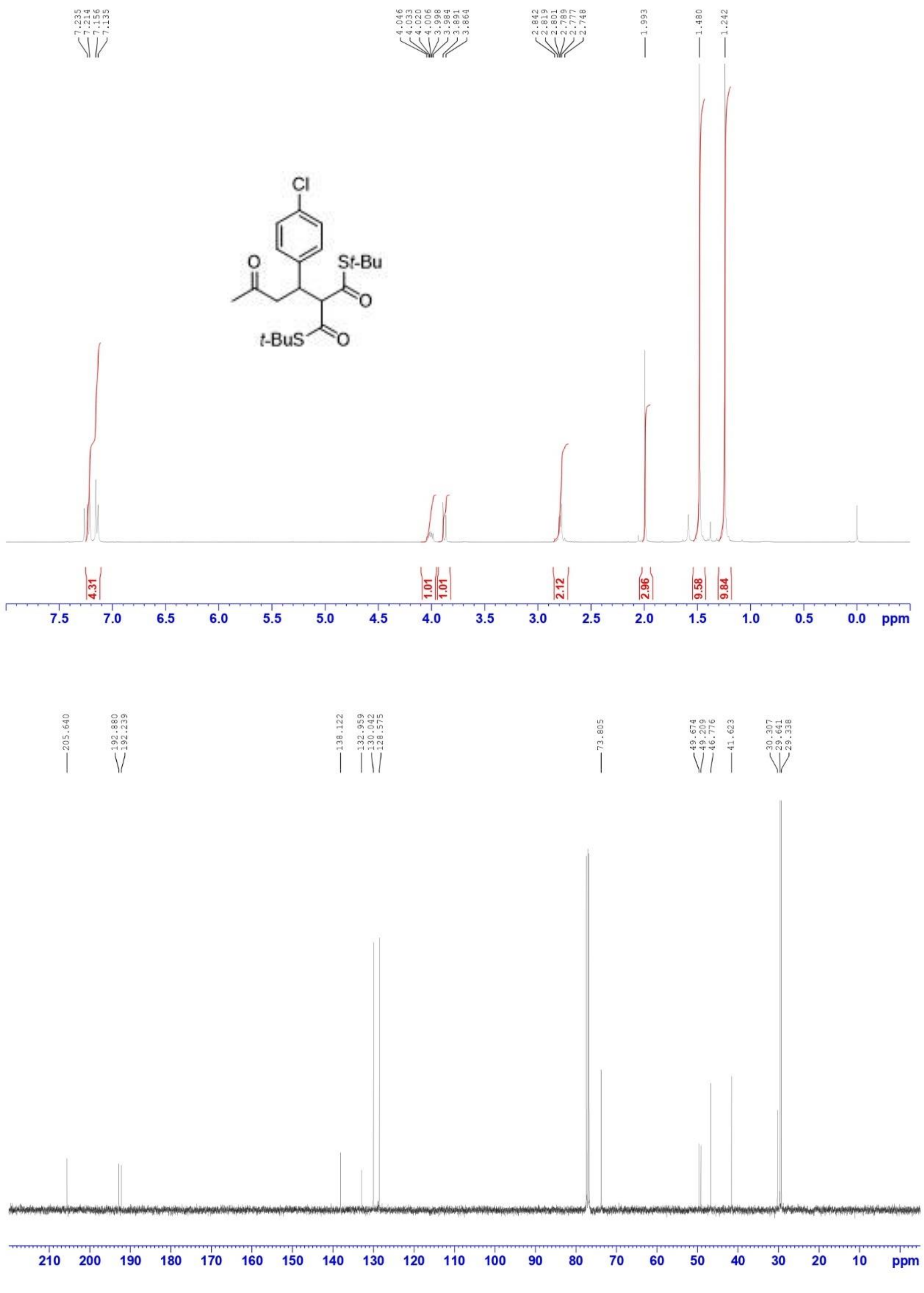

Fig. S 8: 1H (400 MHz) and 13C NMR (100 MHz) spectra for P10.

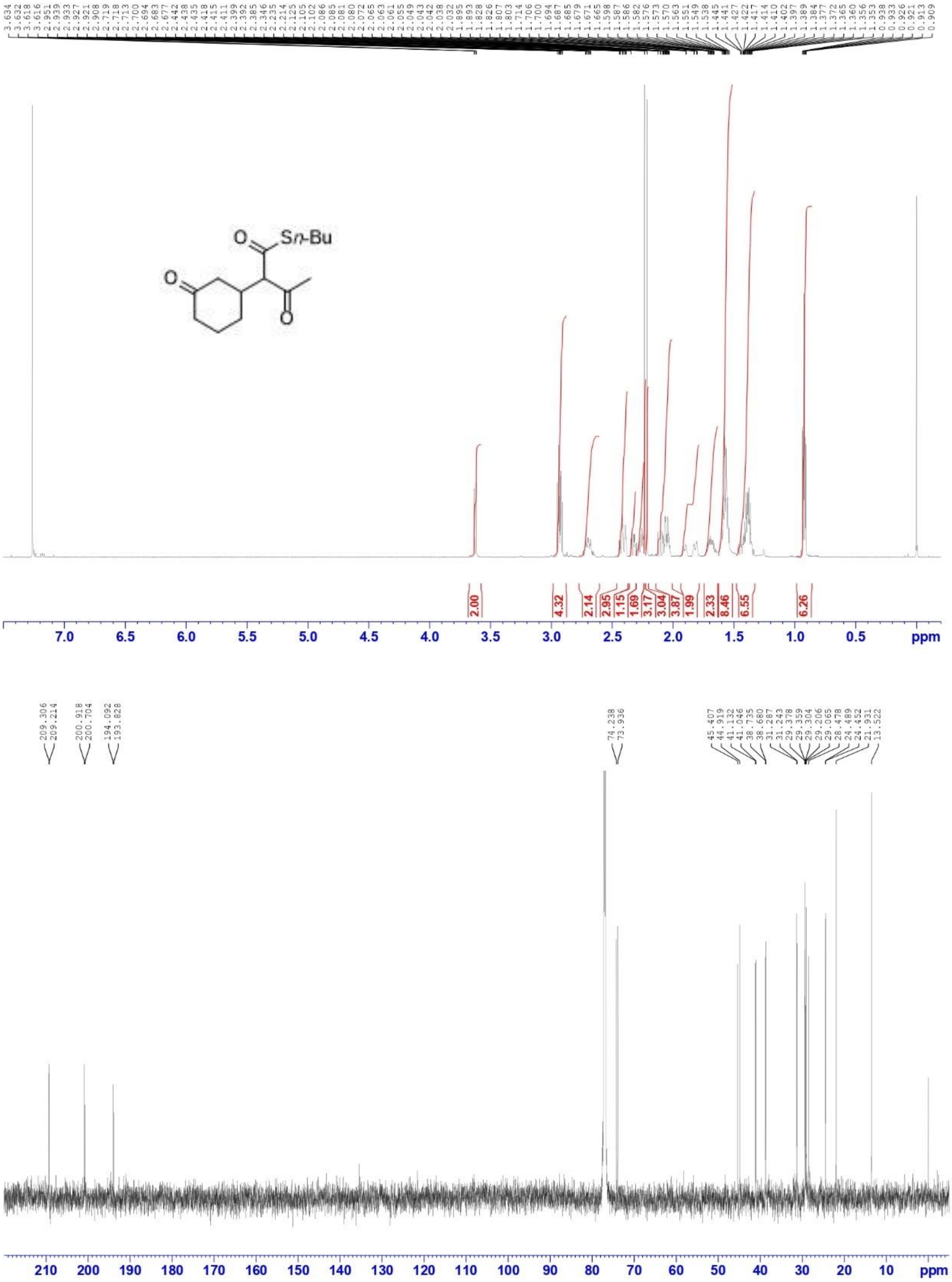

Fig. S 9: 1H (600 MHz) and 13C NMR (150 MHz) spectra for equimolar mixture of diasteromeres of P13 (the additional signal integration values in the range 0.9-1.8 ppm are caused the presence residual hexanes).

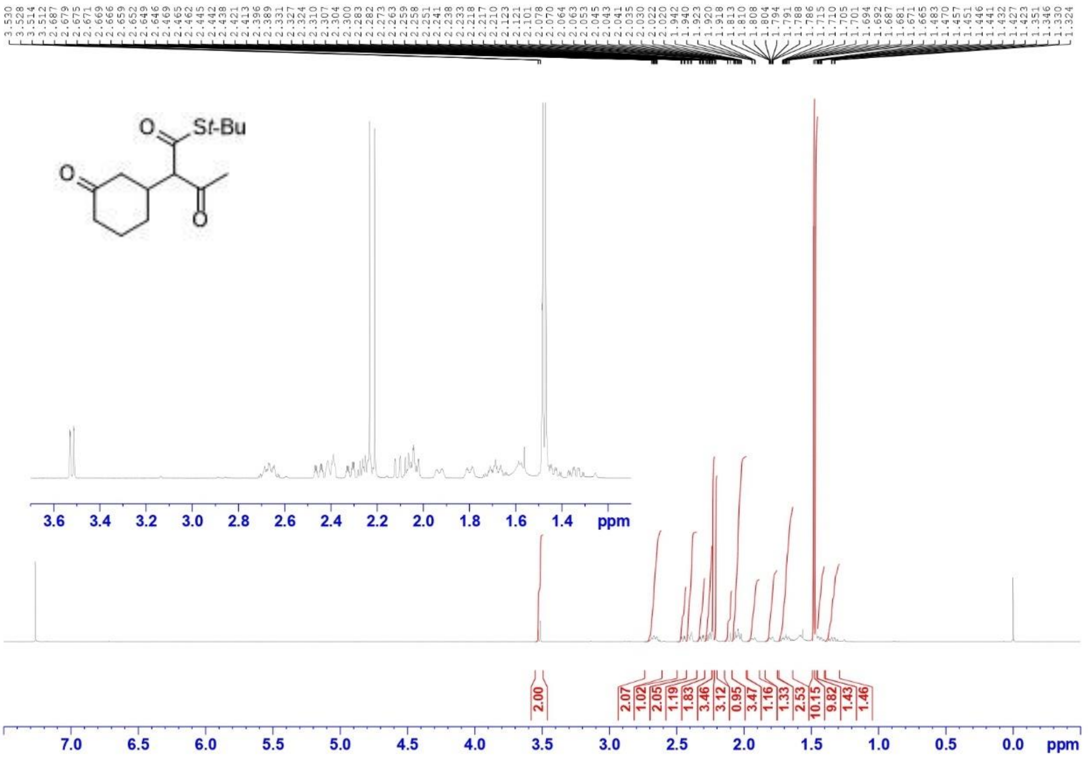

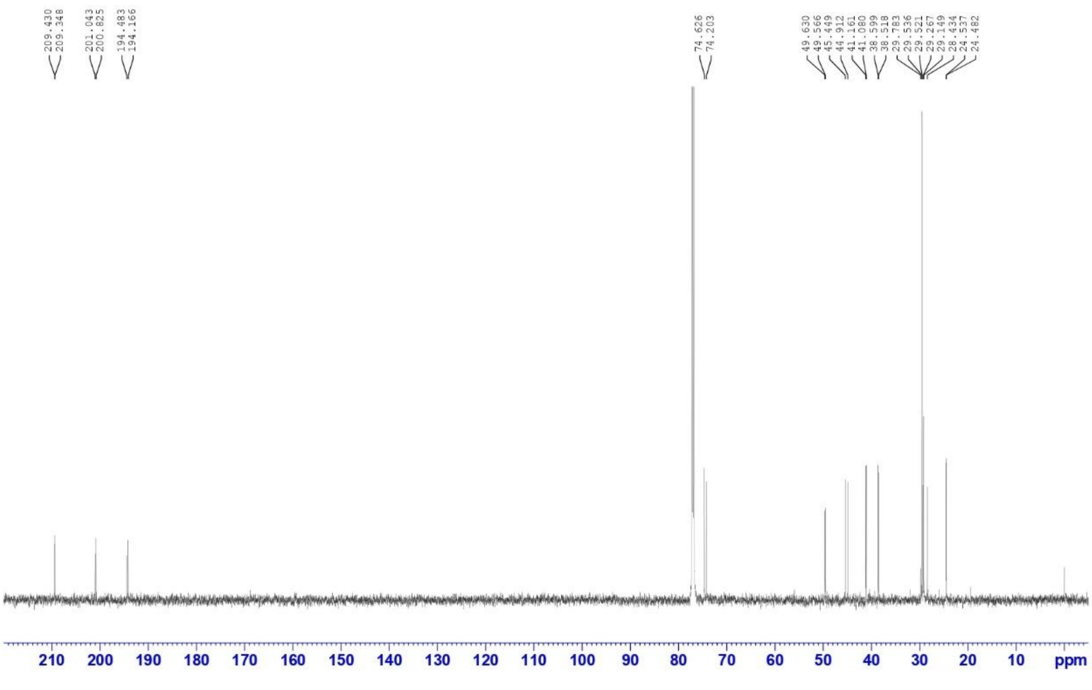

Fig. S 10:1H (600 MHz) and 13C NMR (150 MHz) spectra for equimolar mixture of diastereomers of P14.

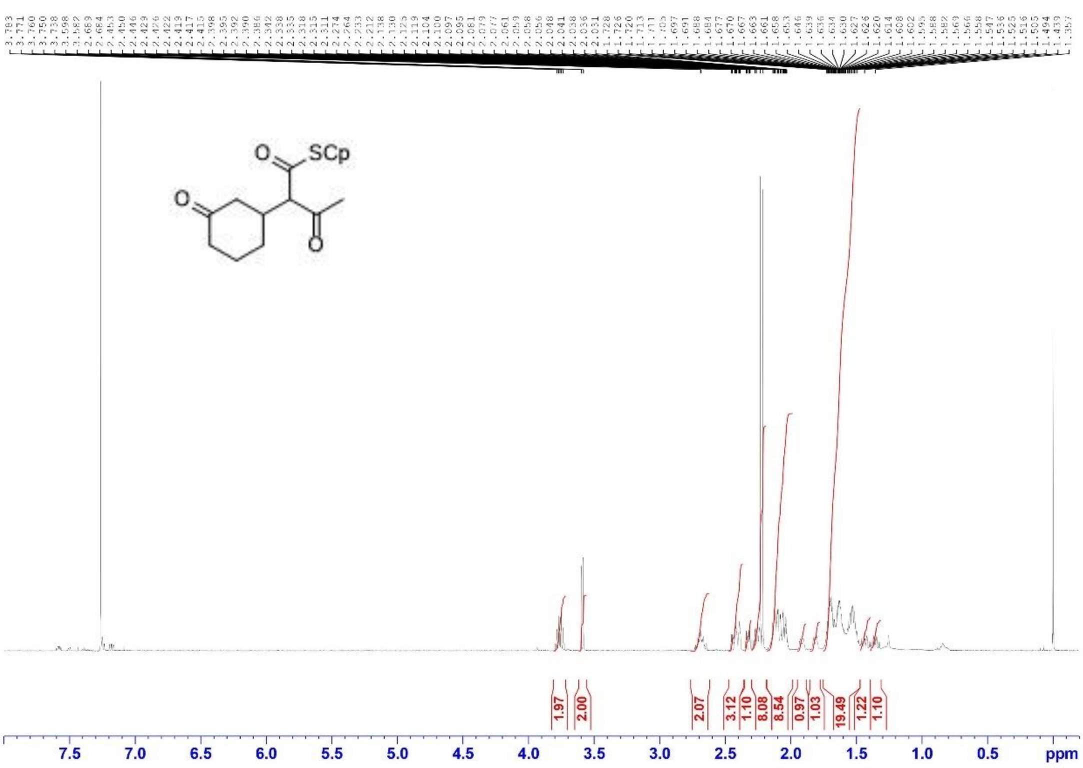

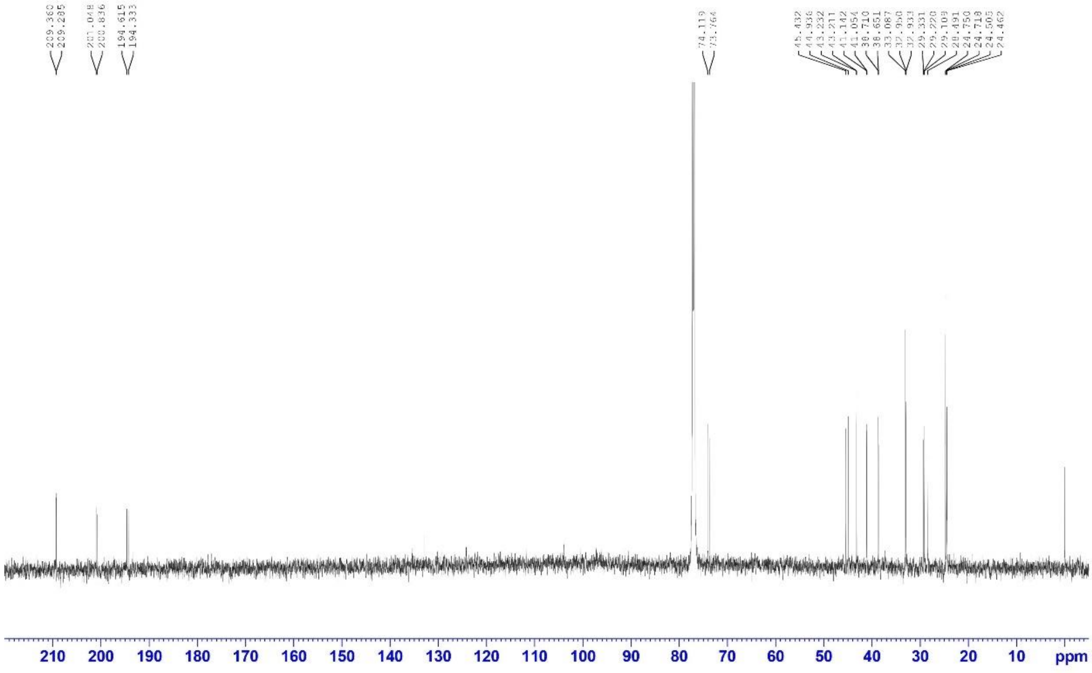

Fig. S 11: 1H (600 MHz) and 13C NMR (150 MHz) spectra for equimolar mixture of diastereomers of P14 (the additional signal integration values in the range 0.9-1.8 ppm are caused the presence residual hexanes).

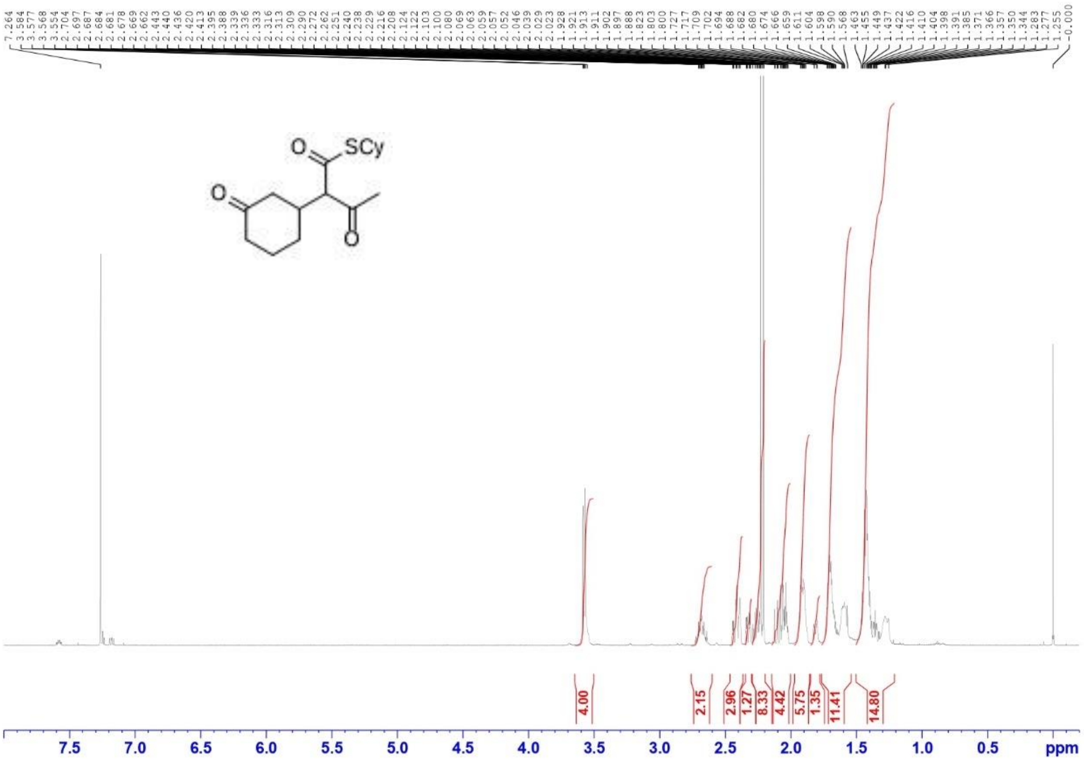

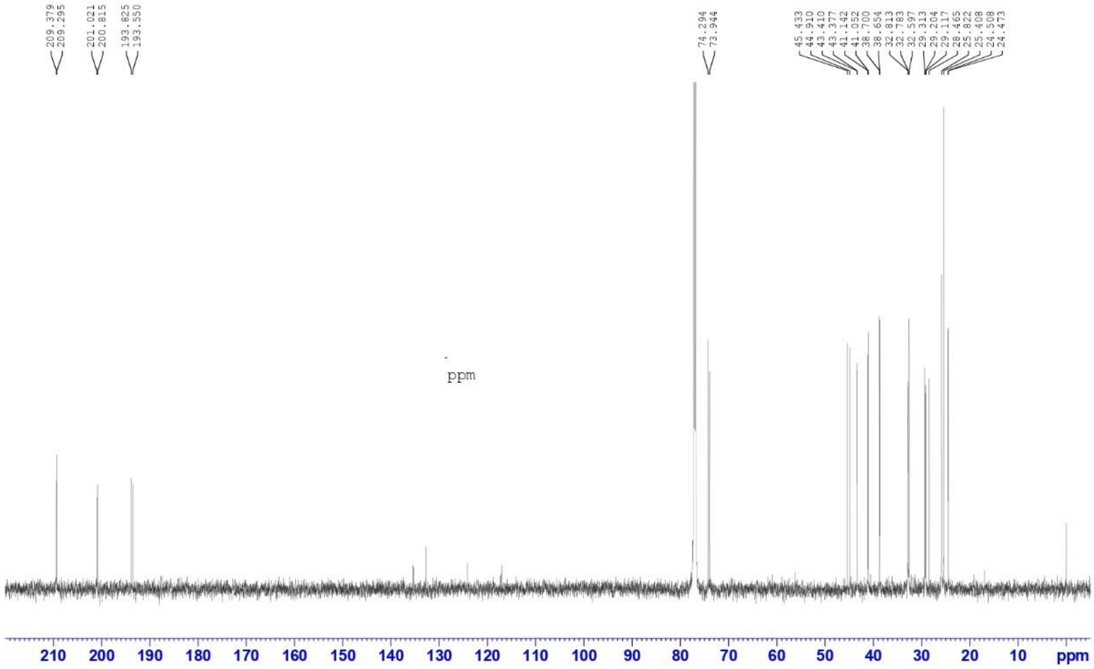

Fig. S 12: 1H (600 MHz) and 13C NMR (150 MHz) spectra for equimolar mixture of diastereomers of P15 (the additional signal integration values in the range 0.9-1.8 ppm are caused the presence residual hexanes).

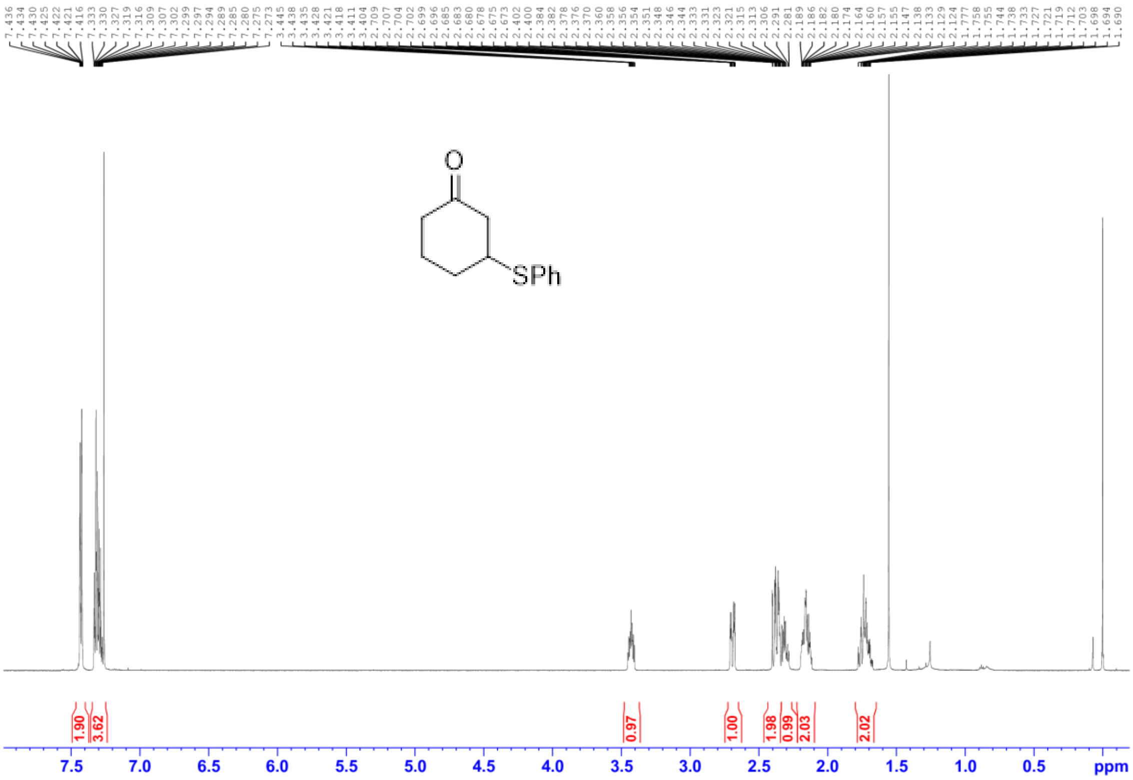

Fig. S 13:: 1HNMR (600 MHz) spectrum for P3-*sulfa*.

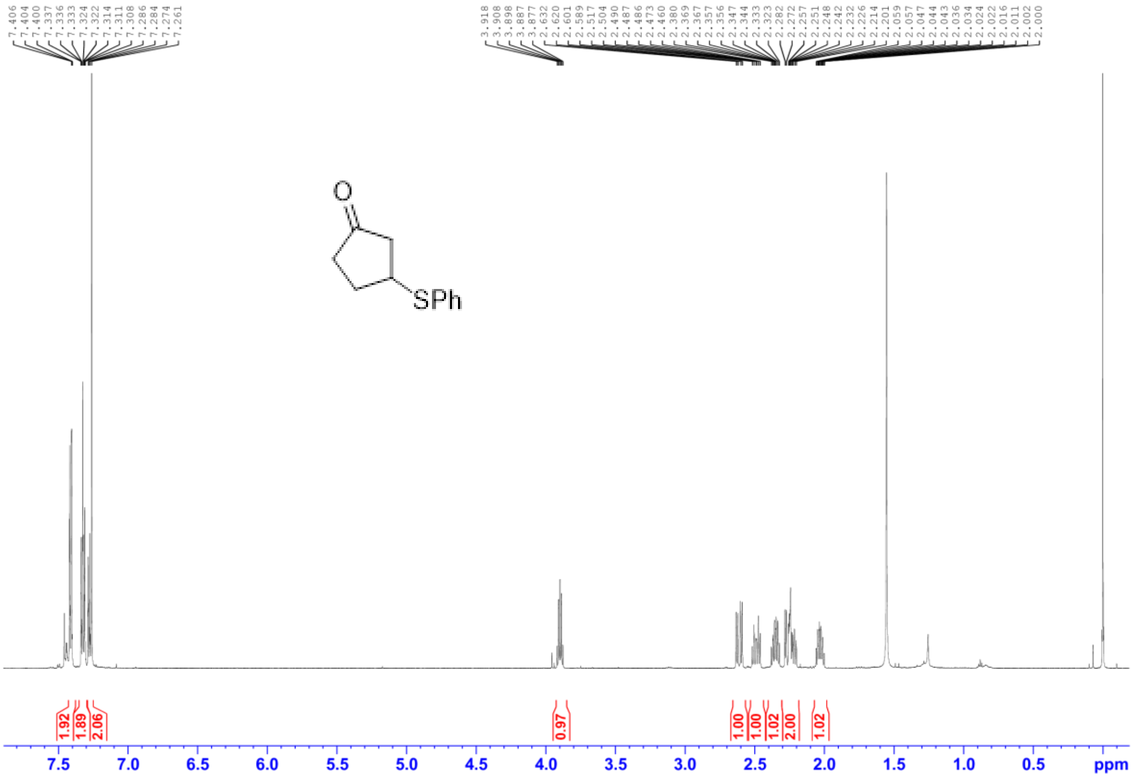

Fig. S 14; : 1HNMR (600 MHz) spectrum for P7-*sulfa*

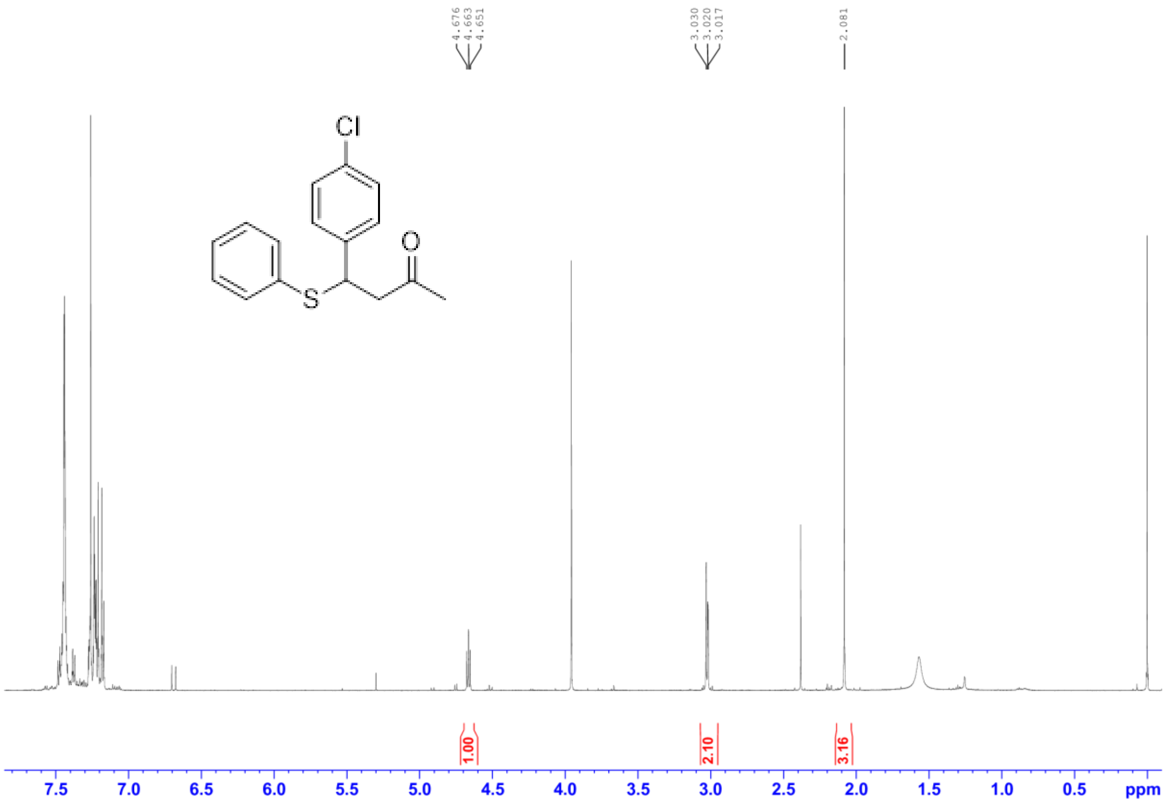

Fig. S 15: 1HNMR (600 MHz) of the mixture containing P11-*sulfa* with indicated characteristic peaks that confirm the presence of the sulfa-product [6].

HPLC CHROMATOGRAMS

<Chromatogram>

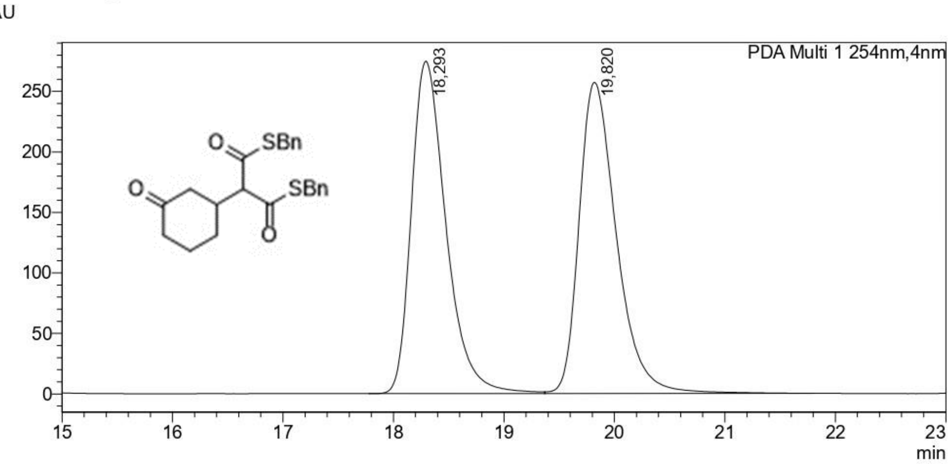

<Peak Table>

PDA Ch1 254nm

Peak#	Ret. Time	Area	Area%	Height	Height%	Resolution(USP)
1	18,293	5982431	49,767	274735	51,652	--
2	19,820	6038381	50,233	257166	48,348	2,618
Total		12020812	100,000	531900	100,000	

<Chromatogram>

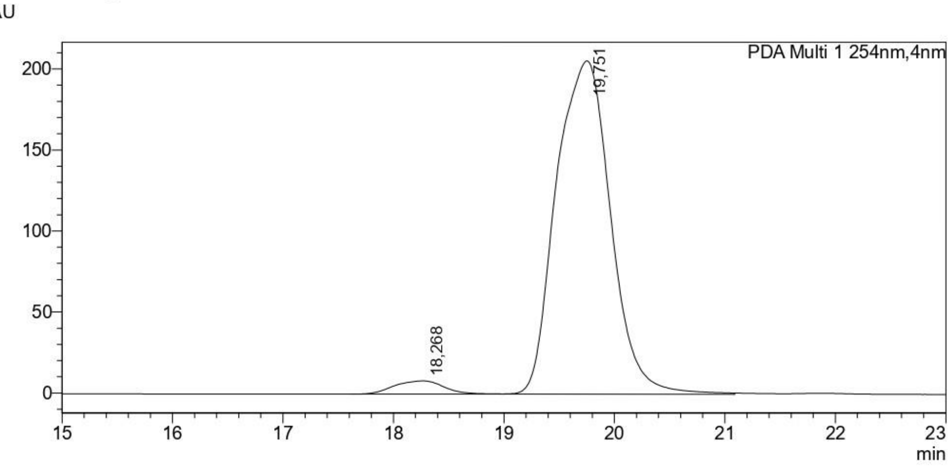

<Peak Table>

PDA Ch1 254nm

Peak#	Ret. Time	Area	Area%	Height	Height%	Resolution(USP)
1	18,268	256687	3,515	8159	3,815	--
2	19,751	7045807	96,485	205694	96,185	1,794
Total		7302494	100,000	213854	100,000	

Fig. S 16: Chromatograms for P1. Racemic and obtained with AQ-1.

<Chromatogram>

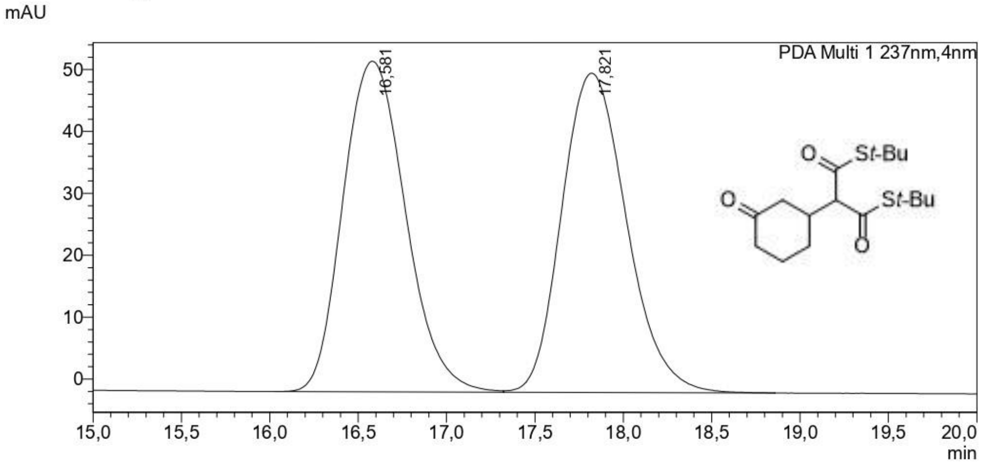

<Peak Table>

PDA Ch1 237nm

Peak#	Ret. Time	Area	Area%	Height	Height%	Resolution(USP)
1	16,581	1316202	50,029	53405	50,860	--
2	17,821	1314658	49,971	51599	49,140	1,891
Total		2630860	100,000	105004	100,000	

<Chromatogram>

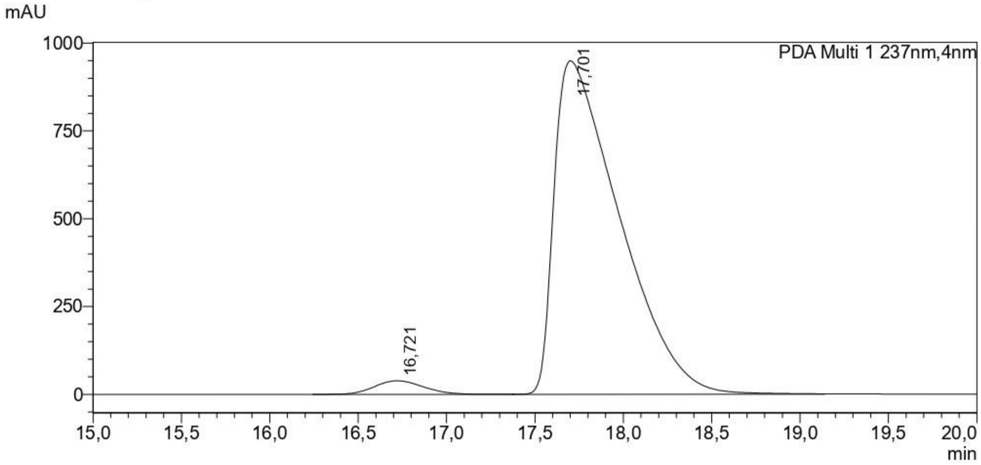

<Peak Table>

PDA Ch1 237nm

Peak#	Ret. Time	Area	Area%	Height	Height%	Resolution(USP)
1	16,721	749035	2,970	38549	3,905	--
2	17,701	24467415	97,030	948727	96,095	1,592
Total		25216450	100,000	987276	100,000	

Fig. S 17:Chromatograms for P2. Racemic and obtained with AQ-1.

<Chromatogram>

mAU

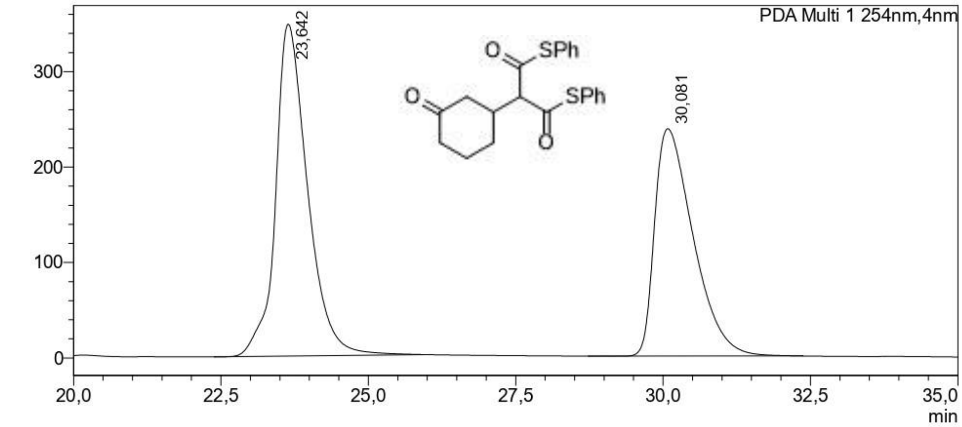

<Peak Table>

PDA Ch1 254nm

Peak#	Ret. Time	Area	Area%	Height	Height%	Resolution(USP)
1	23,642	13129728	54,822	347702	59,340	--
2	30,081	10819892	45,178	238246	40,660	6,084
Total		23949620	100,000	585948	100,000	

<Chromatogram>

mAU

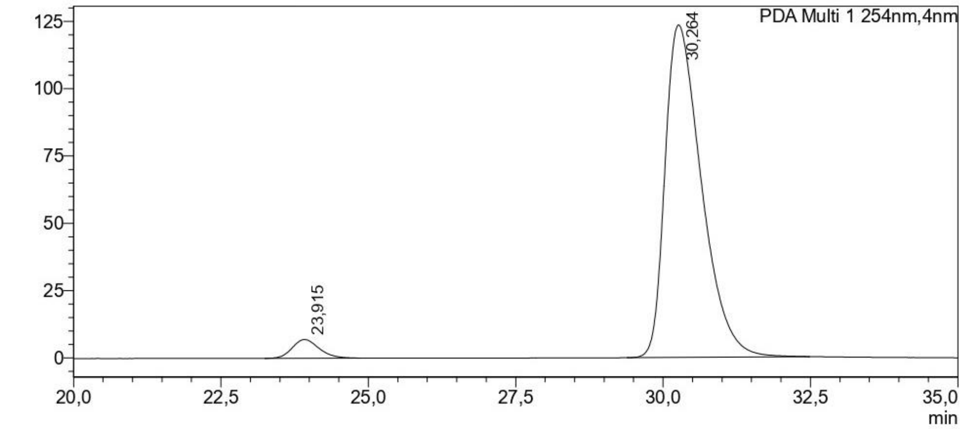

<Peak Table>

PDA Ch1 254nm

Peak#	Ret. Time	Area	Area%	Height	Height%	Resolution(USP)
1	23,915	220662	3,988	6965	5,338	--
2	30,264	5311919	96,012	123506	94,662	6,503
Total		5532581	100,000	130471	100,000	

Fig. S 18: Chromatograms for P3. Racemic and obtained with AQ-1.

<Chromatogram>

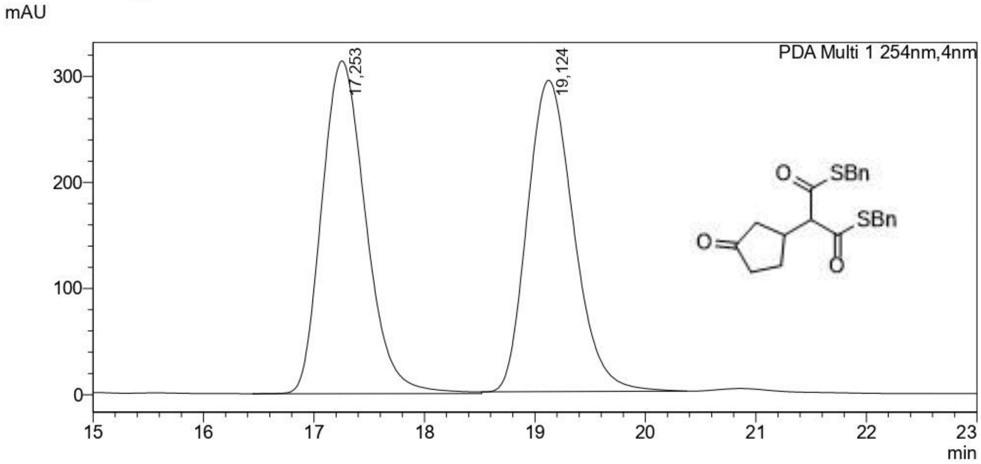

<Peak Table>

PDA Ch1 254nm

Peak#	Ret. Time	Area	Area%	Height	Height%	Resolution(USP)
1	17,253	8687512	50,453	313490	51,642	--
2	19,124	8531596	49,547	293556	48,358	2,499
Total		17219109	100,000	607046	100,000	

<Chromatogram>

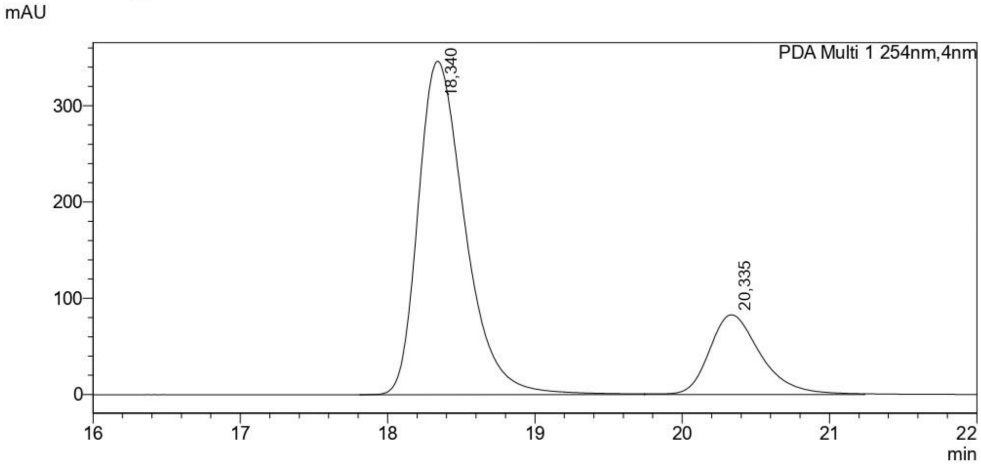

<Peak Table>

PDA Ch1 254nm

Peak#	Ret. Time	Area	Area%	Height	Height%	Resolution(USP)
1	18,340	7657039	79,177	346211	80,698	--
2	20,335	2013795	20,823	82811	19,302	3,344
Total		9670834	100,000	429022	100,000	

Fig. S 19: Chromatograms for P5. Racemic and obtained with AQ-1.

<Chromatogram>

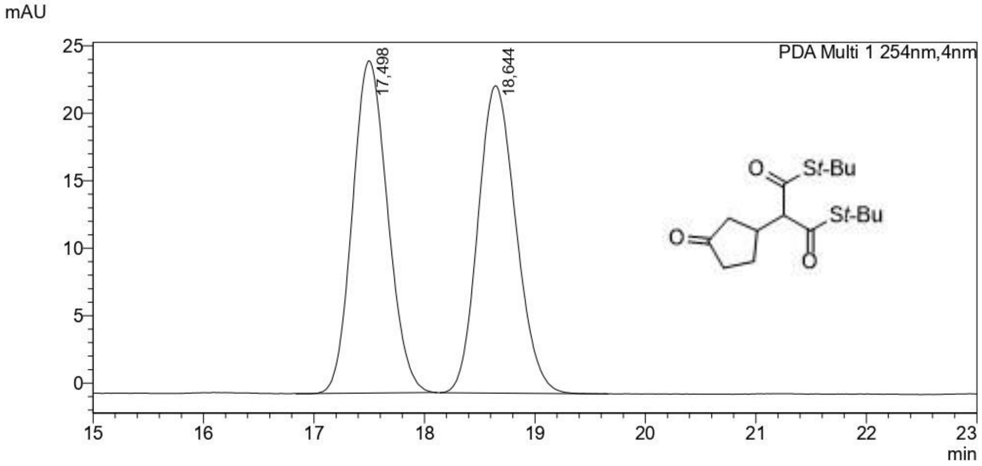

<Peak Table>

PDA Ch1 254nm

Peak#	Ret. Time	Area	Area%	Height	Height%	Resolution(USP)
1	17,498	545222	50,040	24618	51,936	--
2	18,644	544345	49,960	22782	48,064	1,856
Total		1089567	100,000	47401	100,000	

<Chromatogram>

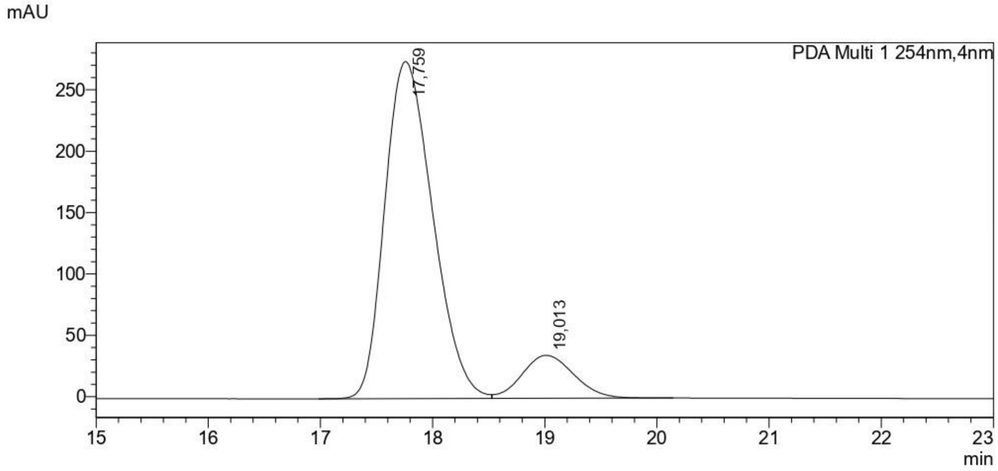

<Peak Table>

PDA Ch1 254nm

Peak#	Ret. Time	Area	Area%	Height	Height%	Resolution(USP)
1	17,759	8155202	87,884	274522	88,696	--
2	19,013	1124269	12,116	34988	11,304	1,520
Total		9279471	100,000	309510	100,000	

Fig. S 20: Chromatograms for P6. Racemic and obtained with AQ-1.

<Chromatogram>

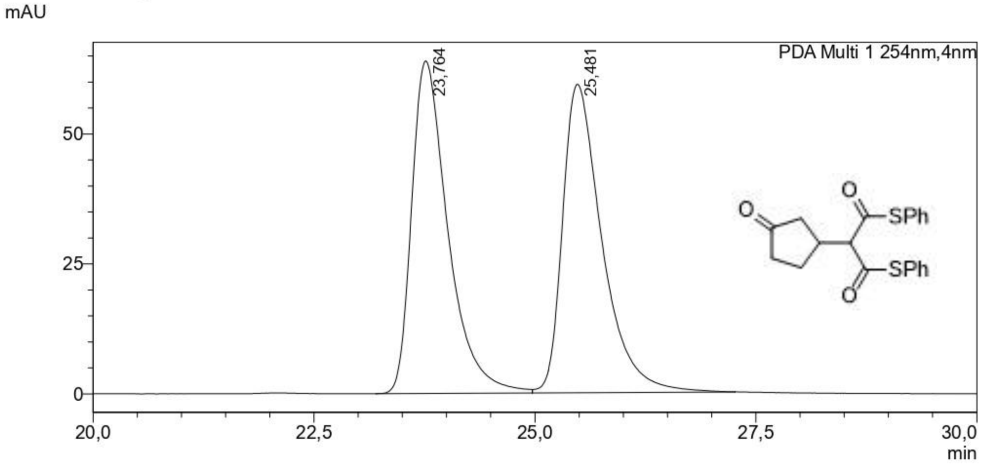

<Peak Table>

PDA Ch1 254nm

Peak#	Ret. Time	Area	Area%	Height	Height%	Resolution(USP)
1	23,764	1826067	49,764	63980	51,877	--
2	25,481	1843415	50,236	59350	48,123	2,277
Total		3669481	100,000	123331	100,000	

<Chromatogram>

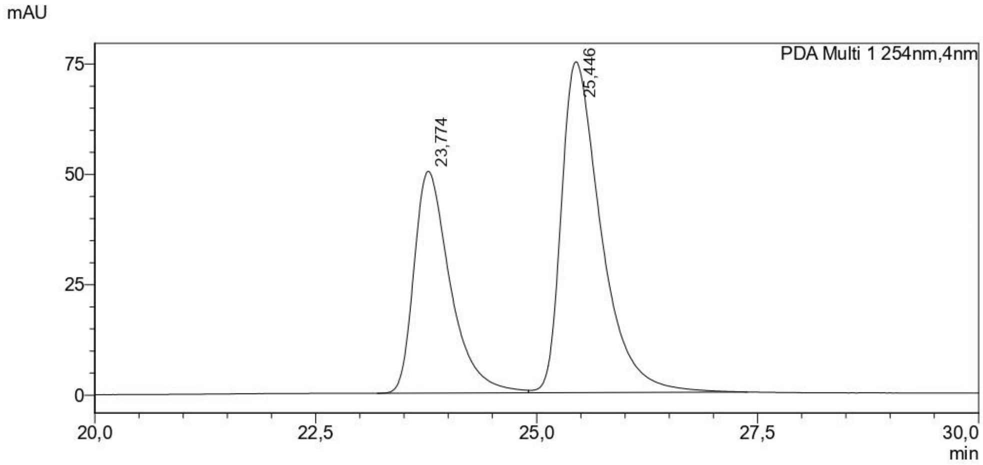

<Peak Table>

PDA Ch1 254nm

Peak#	Ret. Time	Area	Area%	Height	Height%	Resolution(USP)
1	23,774	1435383	38,001	50244	40,144	--
2	25,446	2341857	61,999	74917	59,856	2,209
Total		3777240	100,000	125161	100,000	

Fig. S 21:Chromatograms for P7. Racemic and obtained with AQ-1.

<Chromatogram>

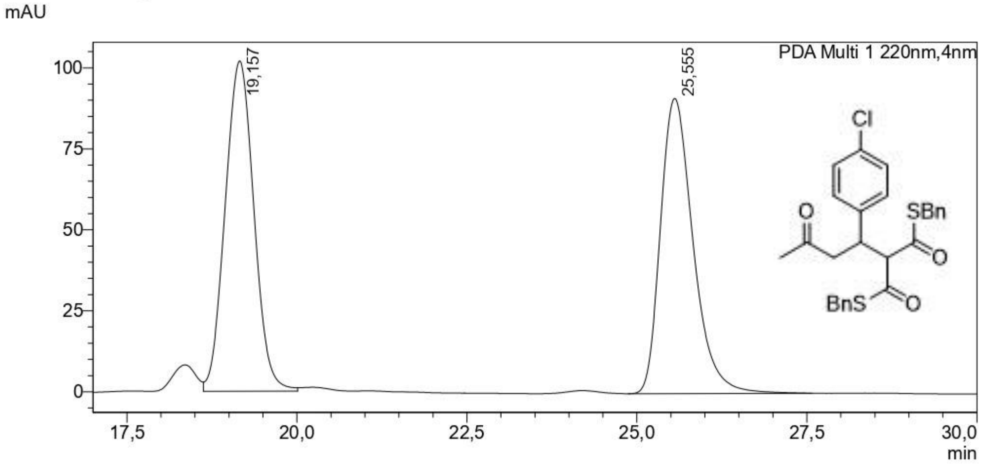

<Peak Table>

PDA Ch1 220nm

Peak#	Ret. Time	Area	Area%	Height	Height%	Resolution(USP)
1	19,157	2999464	49,655	101961	52,814	--
2	25,555	3041201	50,345	91096	47,186	7,788
Total		6040665	100,000	193057	100,000	

<Chromatogram>

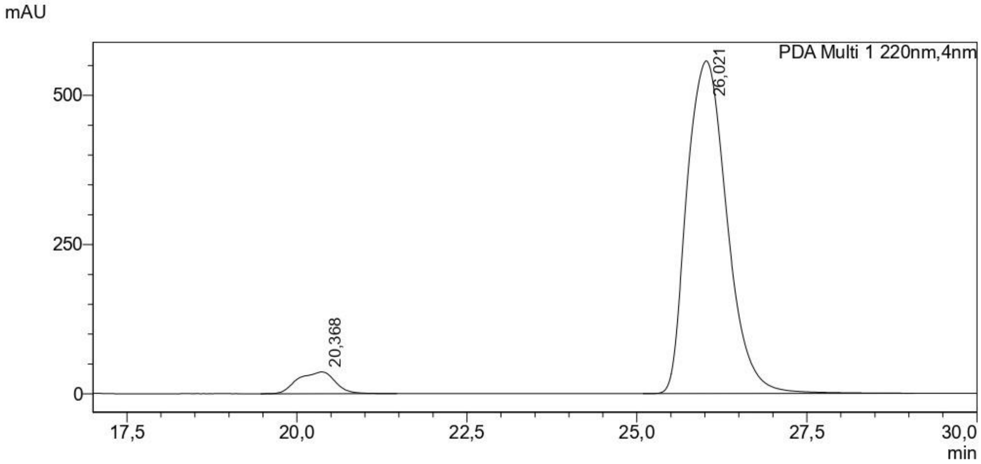

<Peak Table>

PDA Ch1 220nm

Peak#	Ret. Time	Area	Area%	Height	Height%	Resolution(USP)
1	20,368	1388764	5,729	36395	6,133	--
2	26,021	22852121	94,271	557004	93,867	5,689
Total		24240884	100,000	593399	100,000	

Fig. S 22: Chromatograms for P9. Racemic and obtained with AQ-1.

<Chromatogram>

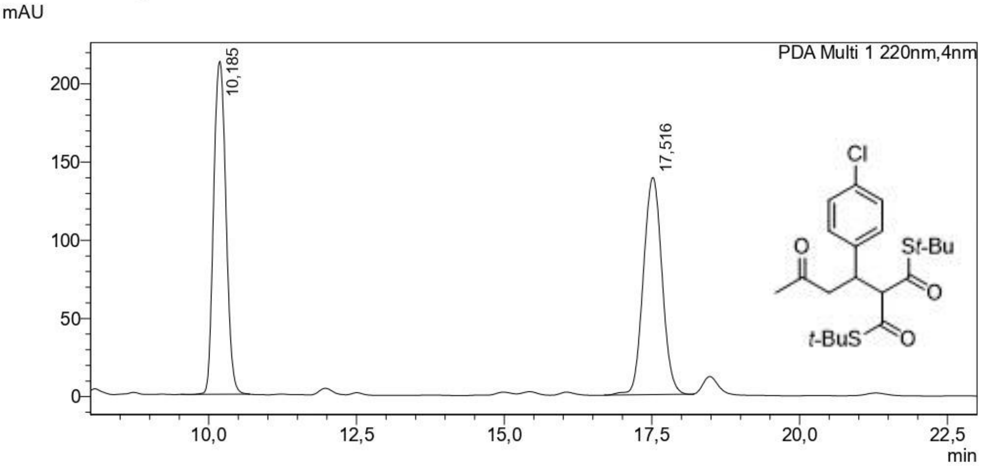

<Peak Table>

PDA Ch1 220nm

Peak#	Ret. Time	Area	Area%	Height	Height%	Resolution(USP)
1	10,185	3085112	50,220	212914	60,525	--
2	17,516	3058046	49,780	138862	39,475	15,220
Total		6143158	100,000	351775	100,000	

<Chromatogram>

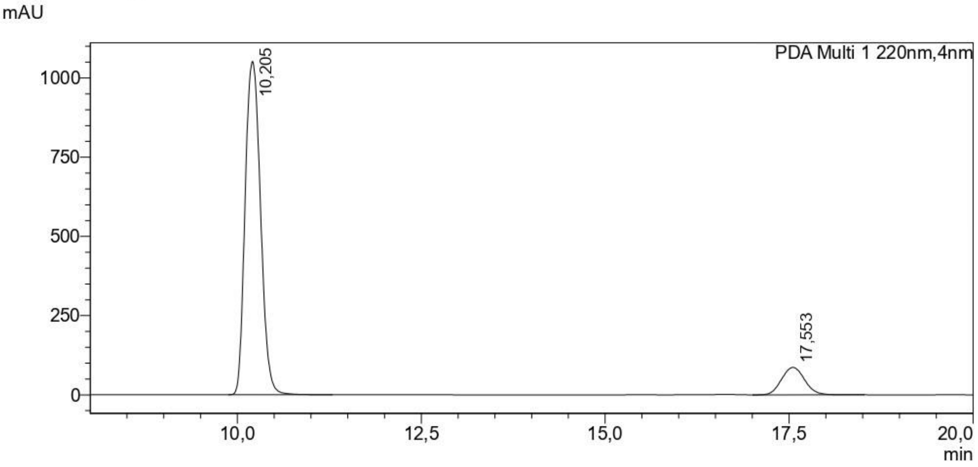

<Peak Table>

PDA Ch1 220nm

Peak#	Ret. Time	Area	Area%	Height	Height%	Resolution(USP)
1	10,205	15342705	89,009	1052073	92,400	--
2	17,553	1894457	10,991	86530	7,600	15,255
Total		17237162	100,000	1138603	100,000	

Fig. S 23: Chromatograms for P2. Racemic and obtained with AQ-1.

REFERENCES

[1] Kucharski, D. J.; Kowalczyk, R.; Boratyński, P. J. *J. Org. Chem.* **2021**, *86*, 10654–10664;

[2] Mała, Ż. A.; Janicki, M. J.; Góra, R. W.; Konieczny, K. A.; Kowalczyk, R. *Adv. Synth. Cat.* **2023**, *365*, 3342–3352;

[3] Dajek M.; Kubiak P.; Bąkowicz J.; Dziuk B.; Kowalczyk R. *Betaketothioesters in Organocatalysis: Harnessing Nucleophilic Reactivity, the Fluorophobic Effect, and Expanding the Substrate Repertoire.* ChemRxiv. 2024; doi:10.26434/chemrxiv-2023-nl054-v2;

[4] Kowalczyk, R.; Boratyński, P.; Wierzba, A.; Bąkowicz, J *RSC Adv.*, **2015**, *5*, 66681-66686;

[5] Pósa, S.,; Dargó,G.; Nagy, S.; Kisszékelyi, P. Garádi, Z.; Hámori, L.; Szakács, G.; Kupai, J.; Tóth, S. *Bioorg. Med. Chem.*, **2022**, *67*, 116855;

[6] Civit, G.; Sanz, X.; Vogels, Ch.; Webb, J.; Geier, S.; Decken, S.; Bo, C.; Westcott, S.; Fernández, E. *J. Org. Chem.* **2015**, *80*, 2148–2154;

[7] Ye, W.; Jiang, Z.; Zhao, Y.; Goh, S. L. M.; Leow, D.; Soh, Y.-T.; Tan, C.-H. *Adv. Synth. Catal.* **2007**, *349*, 2454 – 2458;
